# Histone Lactylation Couples FSH-Driven Lactate Metabolism to Mitochondrial Biogenesis by Enhancing HDAC4-Mediated Deacetylation of PGC-1α in Granulosa Cells

**DOI:** 10.34133/research.1045

**Published:** 2026-01-15

**Authors:** Gang Wu, Min Chen, Chengyu Li, Mengli Wei, Yitong Pan, Tong He, Zhaojun Liu, Hongmin Li, Chunlei Zhang, Jia-Qing Zhang, Yanan Sheng, Yang Liu, Honglin Liu, Ming Shen

**Affiliations:** ^1^ College of Animal Science and Technology, Nanjing Agricultural University, Nanjing 210095, China.; ^2^ State Key Laboratory of Meat Quality Control and Cultured Meat Development, Ministry of Science and Technology (MOST), Nanjing Agricultural University, Nanjing 210095, China.; ^3^ Institute of Animal Husbandry and Veterinary Science, Henan Academy of Agricultural Sciences, Zhengzhou 450002, China.

## Abstract

Follicle-stimulating hormone (FSH) coordinates ovarian follicle development by aligning mitochondrial biogenesis with increased metabolic demand. Although FSH is known to stimulate glycolysis in granulosa cells (GCs), the mechanism by which glycolytic flux coupled to mitochondrial biogenesis remains unclear. Here, we demonstrate that histone lactylation functions as a lactate-sensitive epigenetic mediator linking FSH-driven metabolic alterations to mitochondrial biogenesis in GCs. Mechanistically, FSH increases intracellular lactate levels through glycolytic activation, thereby promoting P300/CBP-dependent lactylation of histone H4 at lysine 5 (H4K5la). H4K5la directly enhances HDAC4 expression, and HDAC4 subsequently deacetylates PGC-1α at lysine residues 329/330. Deacetylated PGC-1α cooperates with nuclear respiratory factors NRF1/2 to drive transcription of key mitochondrial regulators (*TFAM*, *TFB1M*, *TFB2M*), ultimately promoting mitochondrial biogenesis. Disruption of the H4K5la/HDAC4/PGC-1α axis markedly impaired mitochondrial biogenesis and follicular development, evidenced by reduced ovarian weight, smaller follicle size, decreased antral follicle number, and impaired GC proliferation and estradiol (E2) production in FSH-treated mice. These findings identify a metabolic–epigenetic regulatory pathway in which histone lactylation links glycolysis to mitochondrial adaptation, providing mechanistic insight into FSH-dependent reproductive physiology.

## Introduction

Successful folliculogenesis requires precise coordination between endocrine signaling and cellular energy metabolism, and disruption of this coordination is a major cause of ovulatory dysfunction. Granulosa cells (GCs), which support and nurture the developing oocyte, carry out energy-intensive processes, including steroidogenesis [1], proliferation [2], and oocyte maturation support [3]. To meet these demands, GCs dynamically reprogram metabolic pathways and mitochondrial activity: Glycolysis provides rapid adenosine triphosphate (ATP) and biosynthetic intermediates [4], while mitochondrial oxidative phosphorylation sustains long-term energy production and generates essential biosynthetic cofactors [5,6]. Follicle-stimulating hormone (FSH) is the principal endocrine cue driving follicular growth and GC differentiation [7]. In addition to activating canonical follicle-stimulating hormone receptor (FSHR)–adenosine 3′,5′-monophosphate (cAMP)/protein kinase A (PKA) signaling [8], FSH has emerged as a potent metabolic regulator that increases glucose uptake and glycolytic flux in GCs [4], both of which are metabolic changes that are essential for follicle development and oocyte competence.

L-Lactate (lactate), the terminal product of glycolysis, has been traditionally regarded as a metabolic substrate that supplies carbon to mitochondria via conversion to pyruvate and subsequent activation of pyruvate dehydrogenase, fueling the tricarboxylic acid (TCA) cycle and oxidative phosphorylation [9]. However, lactate is now recognized as a bioactive metabolite that modulates redox balance [10], calcium signaling [11], and transcriptional programs in multiple tissues [12]. Recent studies show that lactate also serves as a donor for histone lactylation, a newly described posttranslational modification in which lactate-derived groups are enzymatically added to histone lysine residues [13–17]. This modification directly links cellular metabolic states to chromatin structure and gene regulation, influencing processes such as macrophage polarization and tumor metabolic adaptation. Whether lactate-mediated chromatin remodeling couples glycolytic activity to mitochondrial biogenesis in physiological settings such as folliculogenesis remains unknown.

Mitochondrial biogenesis requires coordinated nuclear–mitochondrial communication [18]. Nuclear transcriptional programs by peroxisome proliferator-activated receptor γ coactivator-1-α (PGC-1α), together with nuclear respiratory factors NRF1 and NRF2 (GABPα), regulate expression of nuclear-encoded mitochondrial proteins and mitochondrial transcriptional machinery (*TFAM*, *TFB1M*, and *TFB2M*) [19]. PGC-1α activity is tightly modulated by posttranslational modifications, including phosphorylation [20], methylation [19], and acetylation [21–23], which influence its stability, localization, and coactivator function. For instance, adenosine monophosphate-activated protein kinase (AMPK)–NAD-dependent deacetylase sirtuin-1 (SIRT1) signaling promotes PGC-1α deacetylation and activation to enhance mitochondrial biogenesis [23], whereas methylation events can disrupt PGC-1α–NRF complex formation [19]. Notably, HDAC4, a class IIa histone deacetylase, deacetylates PGC-1α at conserved lysines (K329/K330) [24], although the physiological relevance of this modification in GC mitochondrial biogenesis remains unclear.

Through integrated metabolic profiling, chromatin analysis, and functional perturbations in GCs and mouse models, we identified a previously uncharacterized FSH-dependent metabolic–epigenetic pathway linking glycolysis to mitochondrial biogenesis. We demonstrated that FSH-stimulated glycolysis increases intracellular lactate, which enhances P300/CBP-mediated H4K5 lactylation (H4K5la), is enriched at promoters of metabolic genes, and directly activates HDAC4 transcription. HDAC4-mediated deacetylation of PGC-1α at K329/K330 facilitates NRF1/2 recruitment and transcription of genes essential for mitochondrial DNA (mtDNA) replication and mitochondrial biogenesis. Inhibition of this lactate–H4K5la–HDAC4–PGC-1α axis suppresses mitochondrial biogenesis and impairs follicular development and steroidogenesis. Together, these results establish a mechanistic framework in which metabolite-sensitive chromatin remodeling translates FSH-induced metabolic signals into nuclear programs that tune mitochondrial capacity to the energetic requirements of follicular growth. This mechanism may represent a broader principle by which metabolic flux controls epigenetic regulation of organelle biogenesis in physiological and pathological contexts.

## Results

### FSH-induced mitochondrial biogenesis is accompanied by elevated H4K5la in ovarian GCs

**Fig. 1.**
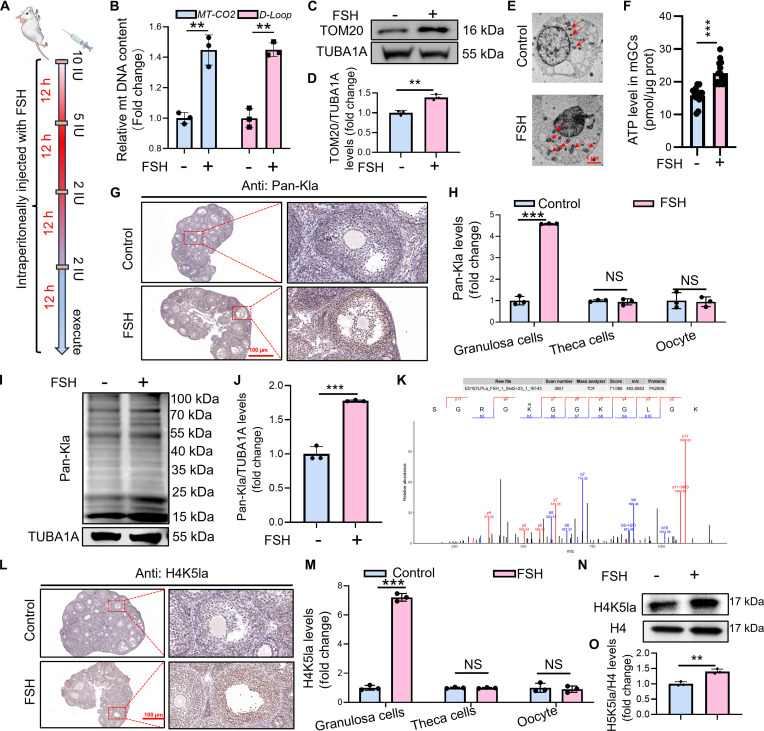
FSH promotes mitochondrial biogenesis in vivo and is associated with elevated H4K5la in ovarian GCs. (A) Schematic diagram illustrating the experimental protocol for intraperitoneal injection of FSH. In brief, mice received intraperitoneal injections of FSH (dissolved in 0.9% saline) every 12 h according to a tapered dosing regimen: 10 IU, 5 IU, and 2 doses of 2 IU, after which samples were collected. Control mice were administered an equivalent volume of 0.9% saline via intraperitoneal injection. (B) qRT-PCR examination of mitochondrial DNA (*MT-CO2* and *D-Loop*) replication levels in ovarian GCs (mGCs) under the FSH dosing regimen depicted in (A). *β-Actin* served as the loading control for data normalization. (C) Western blot analysis of TOM20 protein levels in mGCs according to the FSH administration protocol in (A). (D) The expression level of TOM20 in (C) was determined by quantitative analysis, with TUBA1A serving as the internal control for normalization. (E) Mitochondrial morphology and quantity in mGCs were analyzed by TEM according to the FSH administration protocol in (A). Scale bar, 500 nm. (F) The ATP level in mGCs was measured after intraperitoneal injection of FSH, with normalization to the total protein concentration. (G) Immunohistochemical staining for Pan-Kla protein to assess protein levels and cellular localization in ovarian tissues according to the FSH administration protocol in (A). Scale bar, 100 μm. (H) The number of Pan-Kla^+^ in (G) was quantified and normalized to the total number of cells. (I) Protein expression profiling via Western blot for Pan-Kla in mGCs, as per the FSH dosing regimen depicted in (A). (J) The expression level of Pan-Kla in (I) was determined by quantitative analysis, with TUBA1A serving as the internal control for normalization. (K) Collision-induced dissociation (CID) MS analysis of histone H4 modifications. The MS/MS spectrum of the peptide sequence “(Kla)SGRGKGGKGLGK” highlights the H4 lactylation site. (L) Immunohistochemical detection of H4K5la protein levels and localization in ovarian tissue according to the FSH administration protocol in (A). Scale bar, 100 μm. (M) The number of H4K5la^+^ in (L) was quantified and normalized to the total number of cells. (N) Western blot detection of H4K5la levels in mGCs under FSH treatment (A). (O) The expression level of TOM20 in (N) was determined by quantitative analysis, with histone H4 serving as the internal control for normalization. Data are presented as the mean ± SD from at least 3 independent experiments (*n* ≥ 3). Statistical differences between groups were compared by one-way ANOVA followed by LSD post hoc test.

Our previous work demonstrated that FSH promotes mitochondrial biogenesis in GCs cultured under hypoxic conditions [25]. Here, we evaluated whether similar effects occur in mouse ovarian GCs (mGCs) following intraperitoneal FSH injection (Fig. 1A). FSH stimulation increased the copy number of the mtDNA-encoded cytochrome c oxidase II (*MT-CO2*) gene and mtDNA D-loop region (*D-Loop*) in mGCs (Fig. 1B). Moreover, mGCs displayed elevated levels of the mitochondrial marker TOM20 after FSH treatment (Fig. 1C and D). Transmission electron microscopy (TEM) further showed that FSH significantly increased mitochondrial number in mGCs while preserving ultrastructural integrity, including membrane morphology and cristae organization (Fig. 1E). Mitochondrial volume was also increased, suggesting enhanced mitochondrial fusion after FSH exposure (Fig. 1E). Consistently, expression of the mitochondrial fusion proteins MFN1, MFN2, and OPA1 was up-regulated in mGCs after FSH administration (Fig. S1A to G), whereas levels of the fission protein DRP1 and its phosphorylation at Ser^616^ decreased, and phosphorylation at Ser^637^ increased (Fig. S1A to G), indicating suppressed mitochondrial fission. FSH treatment also significantly elevated ATP level in mGCs (Fig. 1F). These in vivo data confirm that FSH effectively initiates mitochondrial biogenesis under physiological conditions.

Previous studies suggest that intracellular lactate accumulation promotes mitochondrial biogenesis and the TCA cycle activity [26,27]. While the role of lactate is established, the underlying mechanisms remain incompletely defined. Because FSH increases glycolysis during follicular development, leading to elevated lactate [4], we assessed whether lactate contributes to FSH-mediated mitochondrial biogenesis. Following FSH injection, mGCs exhibited significantly increased glucose transporter 1 (GLUT1) expression (Fig. S1H) and elevated lactate levels in mGCs (Fig. S1I).

Recent reports suggest that lactate serves as a precursor for protein lactylation, in which lactyl groups are conjugated to lysine residues [13,28,29]. However, whether FSH regulates mitochondrial biogenesis via lactylation is unknown. Immunohistochemistry revealed a pronounced increase in lactylation in ovarian tissue following FSH treatment, predominantly localized to mGCs (Fig. 1G and H). Western blot analysis further confirmed substantial increases in global protein lactylation (Pan-Kla) in mGCs following FSH stimulation (Fig. 1I and J).

To elucidate the regulatory interplay between histone lactylation and the mitochondrial biogenesis pathway, we isolated GCs from FSH-treated mice and performed mass spectrometry (MS) analysis. Our results identified lactylation at the 5th lysine residue of histone H4 (H4K5la) (Fig. 1K). Immunohistochemical staining further revealed pronounced H4K5la enrichment specifically in GCs, whereas oocytes and theca cells exhibited minimal signal (Fig. 1L and M). This cell type-specific pattern was corroborated by Western blot analysis, which demonstrated significant H4K5la up-regulation in ovarian GCs following FSH administration (Fig. 1N and O). These findings indicate that lactate-derived H4K5la may function as a potential epigenetic mediator of FSH-induced mitochondrial biogenesis in mGCs.

### Inhibition of lactate production/H4K5la suppresses FSH-induced mitochondrial biogenesis in GCs

**Fig. 2.**
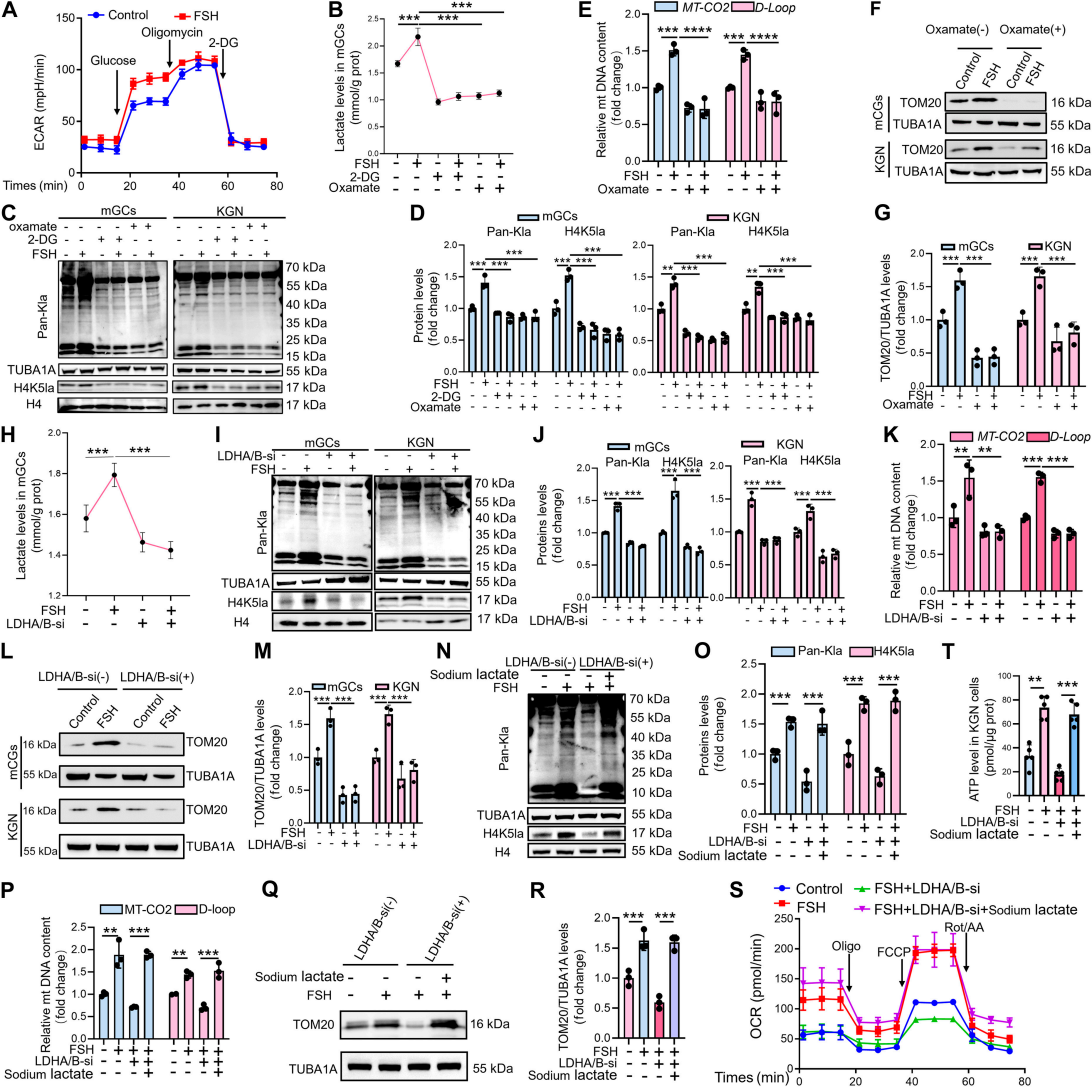
Suppression of lactylation inhibits FSH-induced mitochondrial biogenesis in GCs. (A) ECARs were analyzed in KGN cells with or without FSH treatment. (B) Assessment of intracellular lactate concentrations subsequent to 2 h of 15 mM 2-DG or 15 mM oxamate treatment, and then 12 h of 5-IU FSH administration. Protein concentration served as the normalization control. (C) mGCs/KGN cells after 2-h 2-DG (10 mM) and oxamate (10 mM) pretreatment and then 12-h FSH (5 IU) exposure, and Pan-Kla and H4K5la protein levels were detected by Western blot. (D) Proteins levels in (C) were quantified as follows: Pan-Kla was normalized to TUBA1A, and H4K5la was normalized to histone H4. (E) qRT-PCR assessment of mtDNA quantity (*MT-CO2* and *D-Loop*) after 2-h exposure to 10 mM oxamate and subsequent 12-h treatment with 5 IU of FSH. *β-Actin* served as the loading control for data normalization. (F) Assessment of TOM20 protein concentrations via Western blot in mGCs and KGN cells after 2-h 10 mM oxamate exposure, and then subjected to 5 IU of FSH therapy for 12 h. (G) Protein quantification in (F) was performed using TUBA1A for normalization. (H) Following cotransfection with both LDHA siRNA and LDHB siRNA for 12 h, cells were treated with 5 IU of FSH for 12 h, after which intracellular lactate levels were measured. Protein concentration served as the normalization control. (I) mGCs and KGN cells were transfected with LDHA and LDHB siRNA for 12 h, followed by a 12-h treatment with 5 IU of FSH, after which the protein levels of Pan-Kla and H4K5la were analyzed by Western blot. (J) Proteins levels in (I) were quantified as follows: Pan-Kla was normalized to TUBA1A, and H4K5la was normalized to histone H4. (K) Analysis of mtDNA copy counts for *MT-CO2* and *D-Loop* post-siRNA transfection (12 h), and then treated with 5 IU of FSH for another 12 h. *β-Actin* served as the loading control for data normalization. (L) mGCs and KGN cells were first subjected to LDHA/LDHB knockdown for 12 h and then treated with 5 IU of FSH for another 12 h, after which TOM20 protein levels were analyzed by Western blot. (M) The protein levels of TOM20 in (L) were quantitatively analyzed with normalization to TUBA1A. (N) Following a 12-h transfection of KGN cells with LDHA/LDHB siRNAs, the cells were then treated with 5 IU of FSH for another 12 h. This treatment was conducted both with and without the addition of 15 mM sodium lactate. Afterward, we assessed the protein levels of Pan-Kla and H4K5la using Western blot analysis. (O) Proteins levels in (N) were quantified as follows: Pan-Kla was normalized to TUBA1A, and H4K5la was normalized to histone H4. (P) KGN cells underwent LDHA/LDHB siRNA transfection for 12 h followed by 12-h culture with 5 IU of FSH, supplemented either with or without 15 mM sodium lactate. qRT-PCR examination of mtDNA replication gene (MT-CO2 and D-Loop) copy counts. β-Actin served as the loading control for data normalization. (Q) After transfection with LDHA/LDHB siRNA for 12 h, KGN cells were treated with 5 IU of FSH for another 12 h in medium with or without 15 mM sodium lactate. TOM20 levels were then assessed by immunoblotting. (R) The protein levels of TOM20 in (Q) were quantitatively analyzed with normalization to TUBA1A. (S) KGN cells underwent LDHA/LDHB siRNA transfection for 12 h followed by 12-h culture with 5 IU of FSH, supplemented either with or without 15 mM sodium lactate. OCRs were determined. (T) KGN cells underwent LDHA/LDHB siRNA transfection for 12 h followed by 12-h culture with 5 IU of FSH, supplemented either with or without 15 mM sodium lactate. The ATP level was measured. Protein concentration served as the normalization control. Data are presented as the mean ± SD from at least 3 independent experiments (*n* ≥ 3). Statistical differences between groups were compared by one-way ANOVA followed by LSD post hoc test.

To further determine whether FSH promotes mitochondrial biogenesis via the lactate/histone lactylation pathway, we measured extracellular acidification rate (ECAR) in KGN cells following FSH treatment. Consistent with enhanced glycolytic activity, FSH markedly increased ECAR (Fig. 2A). To modulate intracellular histone lactylation, we employed 2 complementary approaches: (a) pharmacological inhibition of glycolytic flux using 2-deoxy-d-glucose (2-DG) [30,31] and oxamate [32,33] to suppress lactate production, and (b) small interfering RNA (siRNA)-mediated knockdown of lactate dehydrogenase A (LDHA) and LDHB. Both inhibitor treatments effectively reduced intracellular lactate level in mGCs (Fig. 2B) and markedly decreased global protein lactylation as well as H4K5la levels in FSH-stimulated mGCs and KGN cells (Fig. 2C and D). Consistent with these findings, immunofluorescence demonstrated that 2-DG and oxamate efficiently suppressed FSH-induced H4K5la in KGN cells (Fig. S1J to M). Moreover, reduction of histone lactylation significantly impaired FSH-induced mitochondrial biogenesis in GCs, as reflected by mitochondrial DNA copy number (Fig. S1N and Fig. 2E), TOM20 protein abundance (Fig. S1O and P and Fig. 2F and G), and MitoTracker Green fluorescence (Fig. S1Q to T).

In light of the potential off-target effects of 2-DG and oxamate that extend beyond their interference with lactate metabolism, we next genetically depleted LDHA/B to specifically modulate lactate-dependent histone lactylation. Knockdown efficiency is shown in Fig. S2A and B. Dual LDHA/B silencing significantly reduced intracellular lactate generation (Fig. 2H), global protein lactylation (Fig. 2I and J), and H4K5la (Fig. 2I and J). Immunofluorescence analysis confirmed impaired FSH-stimulated H4K5la in KGN cells following LDHA/B knockdown (Fig. S2C and D). Correspondingly, this intervention suppressed FSH-induced mitochondrial biogenesis, as indicated by reduced mitochondrial DNA copy number (Fig. 2K), decreased TOM20 expression (Fig. 2L and M), and diminished MitoTracker Green staining (Fig. S2E and F). To assess whether lactate supplementation could rescue this phenotype, we treated LDHA/B-depleted KGN cells with sodium lactate. Remarkably, exogenous lactate restored H4K5la and mitochondrial biogenesis in FSH-stimulated cells (Fig. 2N to R and Fig. S2G and H). Furthermore, FSH treatment significantly enhanced oxygen consumption rate (OCR) in KGN cells (Fig. 2S), whereas inhibition of lactate production blunted this effect (Fig. 2S); lactate supplementation fully rescued OCR (Fig. 2S). Similarly, LDHA/B knockdown reduced FSH-stimulated ATP production, which was restored by sodium lactate (Fig. 2T). As the key source of energy production in eukaryotic cells, mitochondria undergo coupled oxidative phosphorylation that dynamically modulates membrane potential. Using the JC-1 fluorescent probe to monitor mitochondrial membrane potential (ΔΨm) in real time, we found that FSH treatment markedly increased red fluorescence (J-aggregates, indicating hyperpolarization) while decreasing green fluorescence (JC-1 monomers, indicating depolarization) in KGN cells (Fig. S2I and J). Importantly, pharmacological inhibition of lactate production significantly attenuated FSH-induced ΔΨm elevation, whereas sodium lactate supplementation fully rescued this pro-polarization effect (Fig. S2I and J). To determine whether FSH enhances mitochondrial content by inhibiting mitophagy, we assessed mitophagy markers. In FSH-treated KGN cells, expression of PINK1/Parkin and LC3-II was increased, while p62 expression decreased, consistent with activated mitophagy flux (Fig. S3A to E). Mitochondria–lysosome colocalization also increased after FSH treatment (Fig. S3F). Together, these results indicate that FSH promotes mitochondrial biogenesis in GCs via the lactate/histone lactylation pathway, rather than by suppressing mitophagy.

### FSH promotes mitochondrial biogenesis via P300/CBP-catalyzed H4K5la

**Fig. 3.**
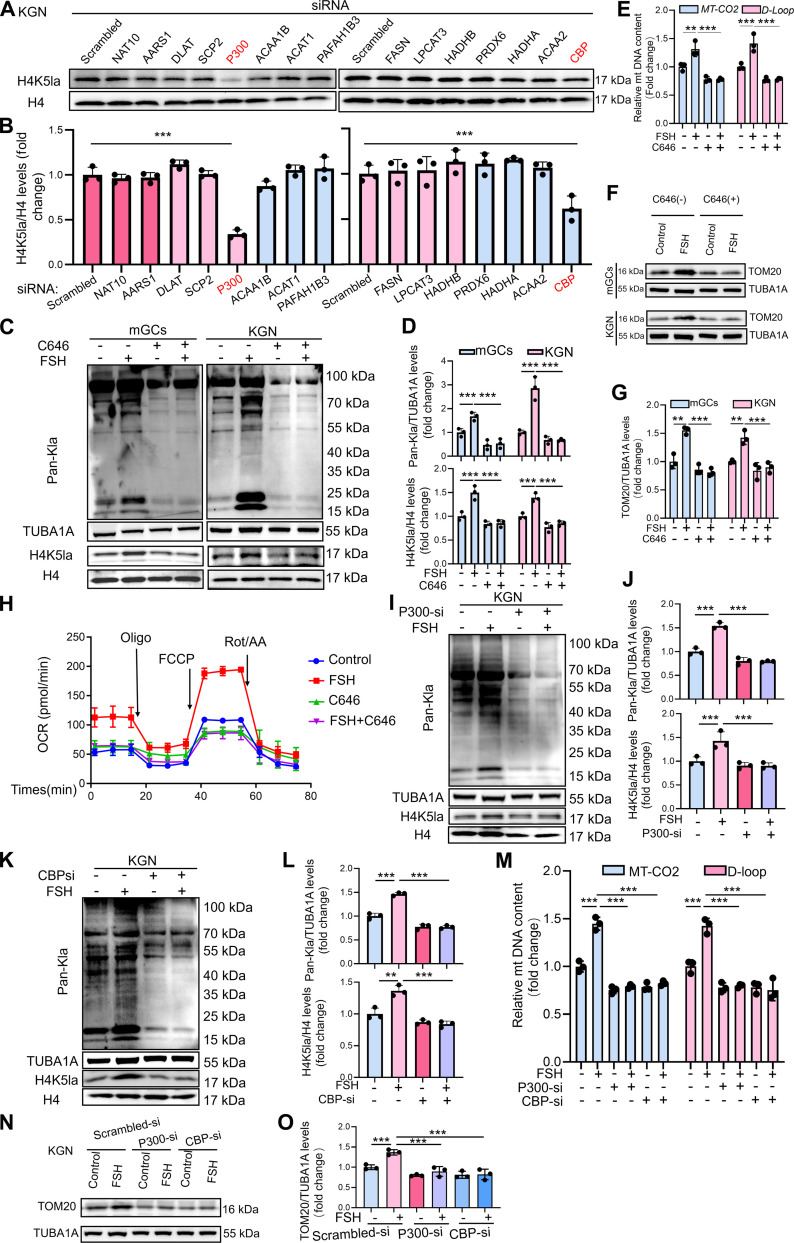
FSH promotes mitochondrial biogenesis in GCs via P300-mediated H4K5la. (A) H4K5la levels were assessed by Western blotting after transfection with acetyltransferase siRNA for 24 h. (B) H4K5la protein levels in (A) were quantitatively analyzed with normalization to H4. (C) Western blot analysis of Pan-Kla and H4K5la protein expression in GCs and KGN treated with 10 μM C646 for 2 h, followed by 5 IU of FSH for 12 h. (D) Protein levels of Pan-Kla and H4K5la in (C) were quantified. Specifically, Pan-Kla was normalized against TUBA1A, and H4K5la was normalized against H4. (E) qRT-PCR analysis of *MT-CO2* and *D-Loop* in KGN treated with 10 μM C646 for 2 h, followed by 5 IU of FSH for 12 h. *β-Actin* served as the loading control for data normalization. (F) Western blot assessing TOM20 expression in GCs and KGN exposed to 10 μM C646 (2 h) and then 5 IU of FSH (12 h). (G) The proteins levels of TOM20 in (F) were quantitatively analyzed with normalization to TUBA1A. (H) KGN cells underwent a 2-h pretreatment with 10 μM C646 and then were exposed to 5 IU of FSH for 12 h, after which OCR was evaluated. (I) Western blot detection of Pan-Kla and H4K5la levels in KGN cells after 12 h of P300 siRNA transfection and subsequent 12-h exposure to 5 IU of FSH. (J) Protein levels of Pan-Kla and H4K5la in (I) were quantified. Specifically, Pan-Kla was normalized against TUBA1A, and H4K5la was normalized against H4. (K) Western blot for Pan-Kla and H4K5la proteins in KGN cells after 12 h of CBP siRNA transfection and subsequent 12-h treatment with 5 IU of FSH. (L) Protein levels of Pan-Kla and H4K5la in (K) were quantified. Specifically, Pan-Kla was normalized against TUBA1A, and H4K5la was normalized against H4. (M) qRT-PCR evaluation of mitochondrial DNA (*MT-CO2* and *D-Loop*) copies in KGN cells posttransfection with P300 or CBP siRNA for 12 h, and then subjected to 5 IU of FSH treatment for an additional 12 h. *β-Actin* served as the loading control for data normalization. (N) Western blot assessing TOM20 expression KGN posttransfection with P300 or CBP siRNA for 12 h, and then subjected to 5 IU of FSH treatment for an additional 12 h. (O) The proteins levels of TOM20 in (N) were quantitatively analyzed with normalization to TUBA1A. Data are presented as the mean ± SD from at least 3 independent experiments (*n* ≥ 3). Statistical differences between groups were compared by one-way ANOVA followed by LSD post hoc test.

To identify the enzyme responsible for H4K5la, we silenced candidate acyltransferases in KGN cells using an siRNA-based approach, with Fig. S4A showing knockdown efficiency. The knockdown of P300 and its homolog CBP depletion reduced H4K5la (Fig. 3A and B), identifying P300/CBP as key lactyltransferases associated with this lactylation signature. We next used C646, a specific P300/CBP antagonist, to inhibit this complex. As expected, C646 treatment effectively suppressed global lactylation and H4K5la in FSH-treated mGCs and KGN cells (Fig. 3C and D). As P300/CBP can also catalyze acetylation by serving as a writer protein, we assessed acetylation levels in FSH-treated KGN cells. However, no significant changes were observed (Fig. S4B and C), and C646 did not eliminate global acetylation (Fig. S4C and D), suggesting that P300/CBP is not the sole acetyltransferase or that its role is compensated in these cells. Next, we examined mitochondrial biogenesis following the inhibition of P300/CBP. Inhibition of P300/CBP abolished FSH-induced increases in mitochondrial DNA content (Fig. 3E) and TOM20 expression (Fig. 3F and G). MitoTracker staining confirmed that C646 blocked FSH-stimulated mitochondrial biogenesis (Fig. S5A and B). Consistent with this, C646 significantly attenuated FSH-stimulated OCR (Fig. 3H) and mitochondrial membrane potential (Fig. S5C and D).

To address the concern that C646 may exert off-target effects beyond lactylation inhibition, we performed individual silencing of P300 and CBP (Fig. S4A). Similarly, loss of enzyme markedly decreased global and H4K5 lactylation (Fig. 3I to L) and proportionally reduced FSH-induced mitochondrial biogenesis in KGN cells, as evidenced by mitochondrial DNA copy number (Fig. 3M), TOM20 expression (Fig. 3N and O), and MitoTracker Green fluorescence (Fig. S5E and F). Knockdown of P300/CBP also suppressed FSH-induced mitochondrial membrane potential elevation (Fig. S5G and H). Collectively, these data demonstrate that P300/CBP-mediated H4K5la contributes to FSH-driven mitochondrial biogenesis in GCs.

### Identification of downstream target genes regulated by H4K5la

**Fig. 4.**
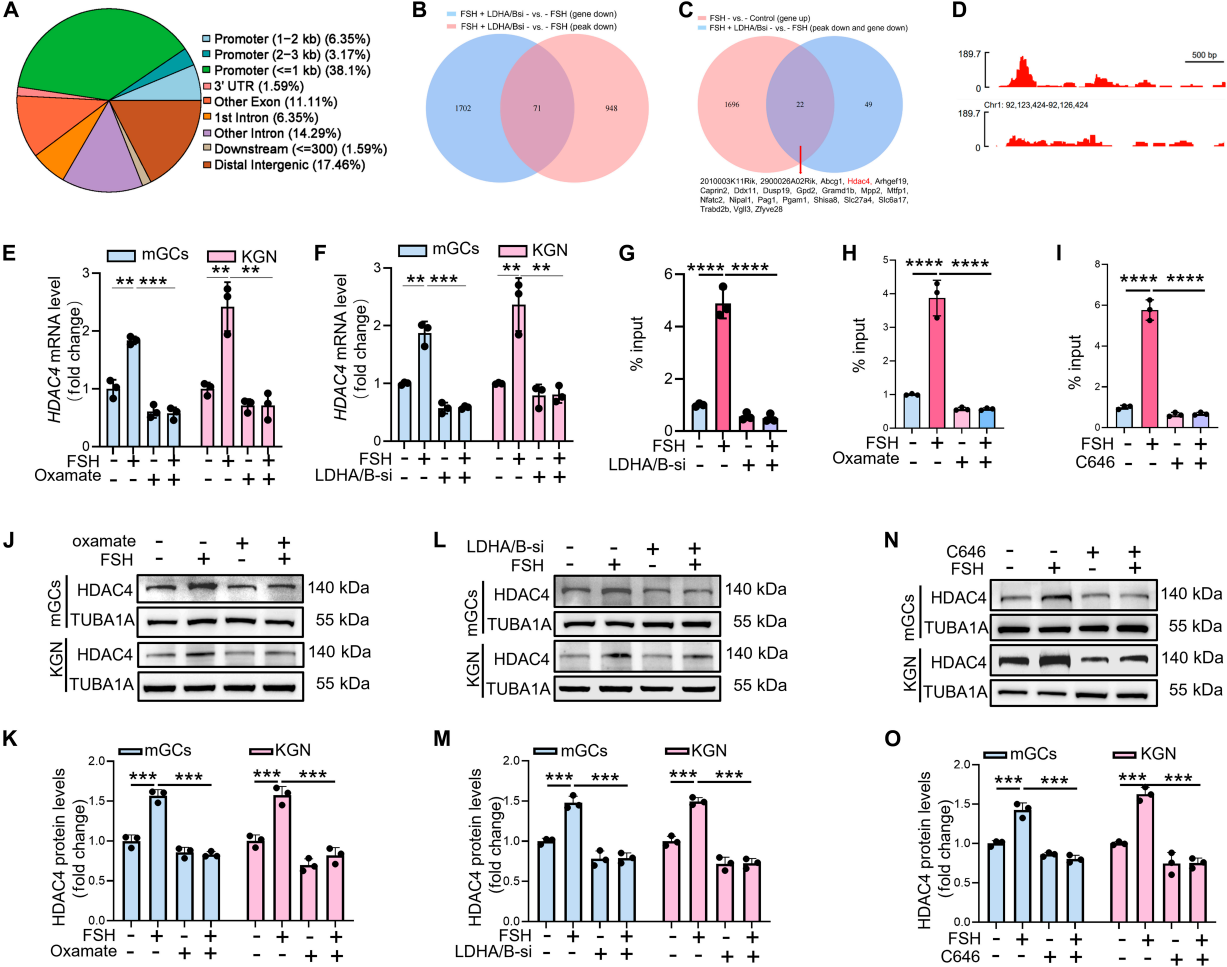
Histone H4K5la activates transcription of HDAC4 in FSH-treated GCs. (A) Elaborate examination of H4K5la binding at various genomic locations within target genes. (B) Strategy for identifying specific downstream targets of H4K5la based on CUT&Tag data. (C) Computational biology suggests that HDAC4 is a possible H4K5la effector. (D) Representative Integrative Genomics Viewer (IGV) tracks showing enriched H4K5la modifications at the HDAC4 promoter in mGCs using CUT&Tag analysis. (E) Assessment of *HDAC4* mRNA abundance via qRT-PCR in mGCs and KGN cells exposed to 15 mM oxamate for 2 h, and then subjected to 5 IU of FSH for 12 h. (F) qRT-PCR was performed to assess *HDAC4* mRNA expression in mGCs and KGN cells after 12-h transfection with LDHA- and LDHB-targeting siRNAs, followed by a 12-h exposure to 5 IU of FSH. (G) ChIP-qPCR revealed H4K5la enrichment at the *HDAC4* promoter in KGN cells pretreated with 15 mM oxamate for 2 h before FSH stimulation (5 IU, 12 h). (H) ChIP-qPCR demonstrated H4K5la binding to the *HDAC4* promoter in KGN cells transfected with LDHA and LDHB siRNAs for 12 h, followed by 5 IU of FSH treatment for an additional 12 h. (I) ChIP-qPCR analysis indicated H4K5la occupancy at the *HDAC4* promoter in KGN cells incubated with 10 μM C646 for 2 h prior to 12-h FSH (5 IU) exposure. (J) Western blotting was used to evaluate HDAC4 protein levels in GCs treated with 15 mM oxamate for 2 h, followed by 5 IU of FSH for 12 h. (K) HDAC4 protein expression in (J) was quantified by densitometry and normalized to TUBA1A as a loading control. (L) Western blot detection of HDAC4 expression in mGCs and KGN cells after 12 h siRNA knockdown of LDHA/LDHB and subsequent 12-h 5-IU FSH exposure. (M) The protein levels of HDAC4 in (L) were quantitatively analyzed with normalization to TUBA1A. (N) Immunoblotting analysis assessing HDAC4 protein abundance in mGCs and KGN cells after 2-h exposure to 10 μM C646, and then 12 h of 5-IU FSH administration. (O) The protein levels of HDAC4 in (N) were quantitatively analyzed with normalization to TUBA1A. Data are presented as the mean ± SD from at least 3 independent experiments (*n* ≥ 3). Statistical differences between groups were compared by one-way ANOVA followed by LSD post hoc test.

To evaluate the impact of histone lactylation on gene expression in FSH-activated GCs, we performed CUT&Tag analysis using an H4K5la-specific antibody. Quality control analyses confirmed high intersample correlation and appropriate DNA fragment size distribution (Fig. S6A and B). H4K5la peaks were predominantly enriched at promoter regions (Fig. S6C and Fig. 4A). Gene oncology (GO) analysis revealed strong enrichment of H4K5la target genes in metabolic regulation in mGCs (Fig. S6D), while Kyoto Encyclopedia of Genes and Genomes (KEGG) pathway annotation similarly demonstrated enrichment in metabolic networks (Fig. S6E), suggesting that histone lactylation contributes to regulation of mitochondrial biogenesis. To examine functional consequences, we conducted RNA sequencing (RNA-seq) on FSH-treated mGCs following LDHA/LDHB knockdown. Differential expression analysis revealed 1,773 down-regulated and 1,335 up-regulated genes in the FSH + LDHA/B-si group compared with FSH-treated controls (Fig. S6F). KEGG enrichment of the down-regulated genes pointed to core metabolic processes (Fig. S6G), indicating that lactate-dependent lactylation supports metabolic gene expression underlying mitochondrial biogenesis. By integrating CUT&Tag data (down-regulated H4K5la peaks) with RNA-seq data (down-regulated genes) from FSH-treated GCs ± LDHA/B knockdown, we identified 71 H4K5la target genes whose transcription was significantly decreased after LDHA/B depletion (Fig. 4B). To further link FSH-induced H4K5la to mitochondrial biogenesis, we analyzed RNA-seq profiles of FSH-treated mGCs, identifying 2,003 down-regulated and 1,718 up-regulated genes (Fig. S6H). KEGG analysis revealed that up-regulated transcripts were closely associated with mitochondrial biogenesis pathways (Fig. S6I). Cross-referencing these genes with the 71 differentially expressed genes with FSH-up-regulated transcripts identified 22 H4K5la target genes that were significantly induced by FSH and markedly down-regulated following LDHA/LDHB knockdown (Fig. 4C). Among these genes, *HDAC4*, which is an HDAC class II deacetylase that acts as an eraser of acetylation but not lactylation, displayed strong H4K5la enrichment at its promoter (Fig. 4D). Consistent with this finding, quantitative real-time polymerase chain reaction (qRT-PCR) confirmed that *HDAC4* mRNA expression was reduced upon oxamate treatment or LDHA/LDHB knockdown in FSH-stimulated mGCs and KGN cells (Fig. 4E and F). Chromatin immunoprecipitation (ChIP)–qPCR further verified H4K5la enrichment at the HDAC4 promoter under FSH stimulation, which was attenuated by oxamate (Fig. 4G), LDHA/B silencing (Fig. 4H), or inhibition of the lactylation writer P300 using C646 (Fig. 4I). Immunoblotting showed decreased HDAC4 protein following oxamate (Fig. 4J and K), LDHA/LDHB knockdown (Fig. 4L and M), or C646 treatment (Fig. 4N and O). Immunofluorescence staining confirmed that inhibiting lactate production and H4K5la reduced HDAC4 protein expression, with HDAC4 localized primarily in the nucleus (Fig. S6J and K). Collectively, these results establish that FSH-induced H4K5la drives transcriptional up-regulation of HDAC4 in GCs.

### HDAC4 contributes to FSH-induced mitochondrial biogenesis

**Fig. 5.**
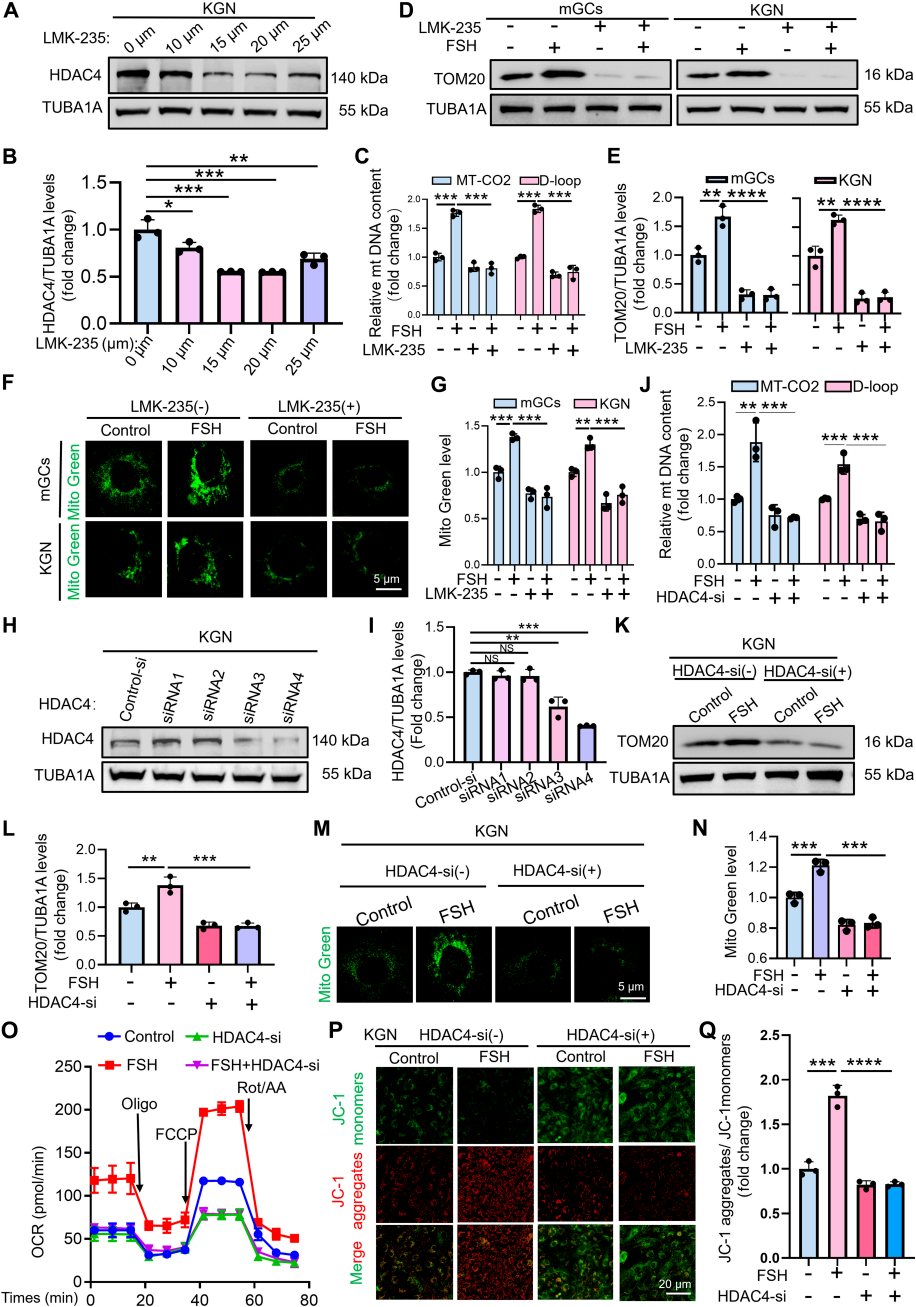
Histone lactylation promotes mitochondrial biogenesis in GCs via HDAC4. (A) Western blot analysis of HDAC4 protein levels in KGN cells cultured with different concentrations of LMK-235 for 12 h. (B) The protein levels of HDAC4 in (A) were quantitatively analyzed with normalization to TUBA1A. (C) qRT-PCR quantification of mitochondrial DNA (*MT-CO2* and *D-Loop*) in KGN cells after 2-h exposure to 15 mM LMK-235, and then 12 h with 5 IU of FSH. *β-Actin* served as the loading control for data normalization. (D) TOM20 protein levels in mGCs and KGN cells were analyzed by Western blot under the following treatment: pretreatment with 15 mM LMK-235 for 2 h, followed by stimulation with 5 IU of FSH for 12 h. (E) TOM20 protein levels in (D) were quantified and normalized to TUBA1A. (F) mGCs and KGN were pretreated with 15 mM LMK-235 for 2 h and then exposed to 5 IU of FSH for 12 h. Mitochondrial labeling was performed using MitoTracker Green (Mito Green) (green), and samples were visualized via confocal microscopy. Scale bar, 5 μm. (G) Quantitative analysis of MitoTracker Green fluorescence intensity from (F). (H) Western blot examination of HDAC4 protein abundance in KGN cells treated with HDAC4 siRNA or control siRNA over a 24-h period. (I) The protein levels of HDAC4 in (H) were quantitatively analyzed with normalization to TUBA1A. (J) qRT-PCR evaluation of mitochondrial DNA copy number in KGN cells post-HDAC4 siRNA transfection for 12 h, and then treated with 5 IU of FSH for another 12 h. *β-Actin* served as the loading control for data normalization. (K) Western blot assessment of TOM20 expression in KGN cells after HDAC4 siRNA transfection (12 h) and subsequent FSH treatment (5 IU, 12 h). (L) The protein levels of TOM20 in (K) were quantitatively analyzed with normalization to TUBA1A. (M) KGN cells received 12 h of HDAC4-specific siRNA transfection and then underwent 12 h of 5-IU FSH treatment. Mitochondria were visualized using MitoTracker Green and imaged by laser confocal scanning microscopy. Scale bar, 5 μm. (N) Quantitative analysis of MitoTracker Green fluorescence intensity from (M). (O) KGN cells received HDAC4-targeting siRNAs for 12 h and then 5 IU of FSH for 12 h. Subsequently, OCR was quantified. (P) KGN cells underwent HDAC4 siRNA transfection for 12 h and then received 5 IU of FSH for another 12 h. JC-1 staining measured mitochondrial membrane potential. (Q) The membrane potential levels in (P) were analyzed. Data are presented as the mean ± SD from at least 3 independent experiments (*n* ≥ 3). Statistical differences between groups were compared by one-way ANOVA followed by LSD post hoc test.

Having established that H4K5la regulates HDAC4 expression, we next investigated the functional role of HDAC4 in FSH-treated GCs. GCs were treated with LMK-235, a specific antagonist of the class II deacetylase family that has been reported to suppress HDAC4 expression at both the transcriptional and translational levels [34]. As expected, HDAC4 expression decreased in a concentration-dependent manner in KGN cells following LMK-235 treatment (Fig. 5A and B). Based on these results, 15 μM LMK-235 was selected as the standard concentration for subsequent experiments. Quantification of mitochondrial DNA content by qRT-PCR (Fig. 5C), immunoblotting of the mitochondrial marker TOM20 (Fig. 5D and E), and MitoTracker Green staining of mitochondria (Fig. 5F and G) demonstrated that pharmacological inhibition of HDAC4 significantly attenuated FSH-induced mitochondrial biogenesis in both mGCs and KGN cells.

To minimize potential off-target effects of LMK-235 unrelated to HDAC4 inhibition, we subsequently employed an siRNA-mediated approach to specifically silence HDAC4. Four siRNAs targeting HDAC4 were initially evaluated for gene silencing efficiency and specificity (Fig. 5H and I). HDAC4 knockdown under FSH stimulation markedly impaired mitochondrial biogenesis, as evidenced by reduced mitochondrial DNA copy number (Fig. 5J), decreased TOM20 protein levels (Fig. 5K and L), and diminished MitoTracker Green fluorescence (Fig. 5M and N). Consistent with these findings, HDAC4 depletion suppressed the FSH-induced increase in both OCR and ΔΨm in KGN cells (Fig. 5O to Q). Collectively, these results firmly establish HDAC4 as a critical regulator of mitochondrial biogenesis in GCs in response to FSH stimulation.

### HDAC4 regulates mitochondrial biogenesis by modulating PGC-1α activity through site-specific deacetylation

**Fig. 6.**
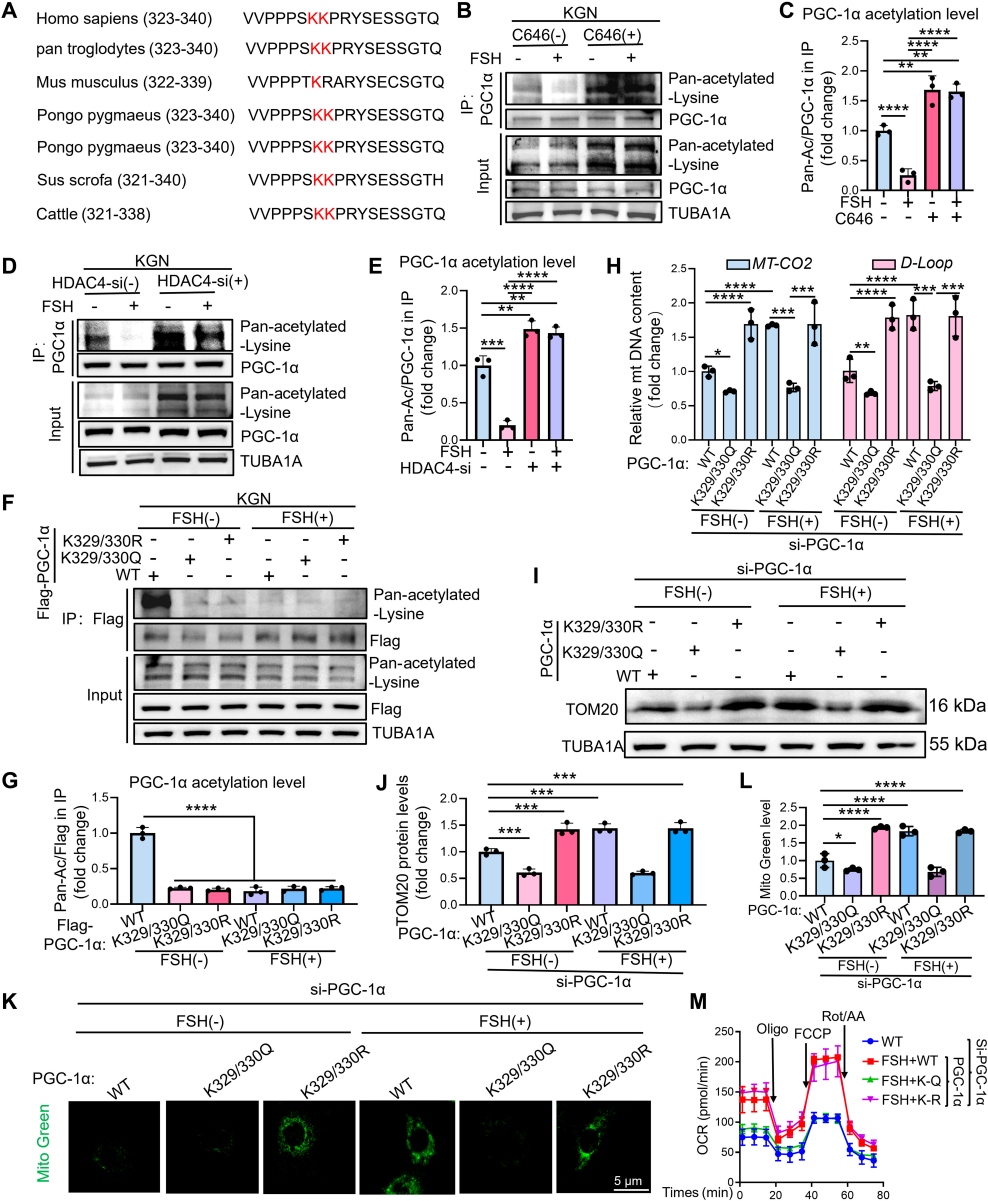
FSH-induced deacetylation of PGC-1α by HDAC4 promotes mitochondrial biogenesis in GCs. (A) Conservation analysis of the PGC-1α K329/330 acetylation site across different species. (B) Analysis by co-IP reveals the engagement of PGC-1α with acetylated lysines, as assessed post-10 μM C646 administration for 2 h and then subjected to 5 IU of FSH exposure for 12 h in KGN cells. (C) Assessment of PGC-1α acetylation levels quantitatively in (B). The level of acetylation was calculated as the ratio of the pan-acetylated-lysine signal to the total PGC-1α signal following co-IP. (D) IP analysis identified the association of PGC-1α with pan-acetylated lysines post-HDAC4 silencing for 12 h, subsequent to 12 h of 5-IU FSH treatment in KGN cells. (E) Quantitative analysis of the acetylation modification level of PGC-1α in (D). The level of acetylation was calculated as the ratio of the pan-acetylated-lysine signal to the total PGC-1α signal following co-IP. (F) IP technique to identify the association of PGC-1α with all acetylated lysines in PGC-1α knockdown KGN cells overexpressing Flag-tagged WT PGC-1α, K329/330R PGC-1α (acetylation-resistant), or K329/330Q PGC-1α (acetylation-mimic) for 12 h, and then treated with 5 IU of FSH for 12 h. (G) Quantitative analysis of the acetylation modification level of PGC-1α in (F). The level of acetylation was calculated as the ratio of the pan-acetylated-lysine signal to the total Flag-PGC-1α signal following co-IP. (H) qRT-PCR analysis of mitochondrial DNA copy number (*MT-CO2* and *D-Loop*) in PGC-1α knockdown KGN cells overexpressing Flag-tagged WT PGC-1α, K329/330R PGC-1α (acetylation-resistant), or K329/330Q PGC-1α (acetylation-mimic) for 12 h. Following transfection, the cells were stimulated with 5 IU of FSH for an additional 12-h period before analysis. *β-Actin* served as the loading control for data normalization. (I) Western blot analysis of TOM20 protein levels in KGN cells overexpressing Flag-tagged WT PGC-1α, K329/330R PGC-1α, or K329/330Q PGC-1α for 12 h, followed by treatment of 5 IU of FSH. (J) The protein levels of TOM20 in (I) were quantitatively analyzed with normalization to TUBA1A. (K) PGC-1α knockdown KGN cells overexpressing Flag-tagged WT PGC-1α, K329/330R PGC-1α (acetylation-resistant), or K329/330Q PGC-1α (acetylation-mimic) for 12 h, followed by 5 IU of FSH for an additional 12 h. MitoTracker Green (green) labeled mitochondria, visualized via laser confocal microscopy. Scale bar, 5 μm. (L) Quantitative analysis of MitoTracker Green fluorescence intensity from (K). (M) PGC-1α knockdown KGN cells overexpressing Flag-tagged WT PGC-1α, K329/330R PGC-1α (acetylation-resistant), or K329/330Q PGC-1α (acetylation-mimic) for 12 h, followed by 5 IU of FSH for an additional 12 h. The OCRs were then measured. Data are presented as the mean ± SD from at least 3 independent experiments (*n* ≥ 3). Statistical differences between groups were compared by one-way ANOVA followed by LSD post hoc test.

PGC-1α is a well-established master regulator of metabolic pathways. Previous studies have demonstrated that HDAC4 directly targets PGC-1α and deacetylates lysine residues K329 and K330 to regulate its activity [24]. However, whether acetylation at these residues influences mitochondrial biogenesis remained unknown. To address this, we examined the role of HDAC4-mediated lysine deacetylation of PGC-1α in mitochondrial regulation. Cross-species sequence alignment revealed strong evolutionary conservation of PGC-1α K329/K330 (Fig. 6A), highlighting their potential functional importance. We first assessed PGC-1α acetylation in FSH-stimulated KGN cells and observed a significant reduction in acetylation following FSH treatment (Fig. 6B and C), which was reversed by the P300/CBP inhibitor C646 (Fig. 6B and C). Similarly, HDAC4 knockdown increased PGC-1α acetylation in FSH-treated cells (Fig. 6D and E).

Next, we mutated the K329/330 residue individually to non-acetylatable arginine (R) residue or to acetylatable glutamine (Q) residue and examined the acetylation levels of each mutant when exposed to FSH. KGN cells were engineered to express either wild-type (WT) Flag-PGC-1α or its acetylation-mimetic (K329/330Q) and non-acetylatable (K329/330R) variants via liposomal transfection. To ensure detection of direct acetylation, immunoprecipitated complexes were dissociated with sodium dodecyl sulfate (SDS) prior to immunoblotting with pan-acetyl-lysine antibody. The K329/330R mutation nearly abolished FSH-induced PGC-1α acetylation, confirming that these sites represent the primary acetylation sites (Fig. 6F and G).

To comprehensively map acetyltransferases associated with PGC-1α, we performed IP followed by MS in NIH/3T3 cells. This approach defined the PGC-1α interactome and identified 1,472 putative interacting proteins, including potential mediators of its acetylation (Fig. S7A). GO enrichment analysis highlighted a strong association between these interactors and mitochondrial biogenesis pathways (Fig. S7B). Among these proteins, 11 candidate acetyltransferases were prioritized based on functional annotation (Fig. S7A). Targeted siRNA screening identified DLAT and ACAT1 as key modulators of PGC-1α posttranslational modification, as their depletion substantially diminished PGC-1α acetylation (Fig. S7C and D). Subsequent co-IP experiments confirmed interaction of both enzymes with PGC-1α, and these interactions were dynamically disrupted by FSH stimulation (Fig. S7E to G).

We then assessed whether PGC-1α acetylation affects FSH-induced mitochondrial biogenesis. KGN cells were transfected with WT PGC-1α or the K329/330R and K329/330Q mutants following siRNA-mediated depletion of endogenous PGC-1α. The efficiency of PGC-1α knockdown is demonstrated in Fig. S4F. The K329/330R mutant (deacetylation-mimetic) significantly enhanced mitochondrial biogenesis (Fig. 6H to L), whereas the acetyl-mimetic K329/330Q mutant impaired it, as reflected by (a) reduced mitochondrial DNA copy number (Fig. 6H), (b) decreased TOM20 levels (Fig. 6I and J), and (c) reduced MitoTracker Green staining (Fig. 6K and L). Correspondingly, the K329/330R mutant elevated mitochondrial OCR (Fig. 6M), ATP levels (Fig. S7H), and ΔΨm (Fig. S7I and J), whereas the K329/330Q mutant exerted the opposite effects (Fig. 6M and Fig. S7I and J). These findings collectively demonstrate that FSH promotes mitochondrial biogenesis via HDAC4-mediated deacetylation of PGC-1α at K329/K330.

### PGC-1α deacetylation recruits NRF1/2 to drive mitochondrial biogenesis

**Fig. 7.**
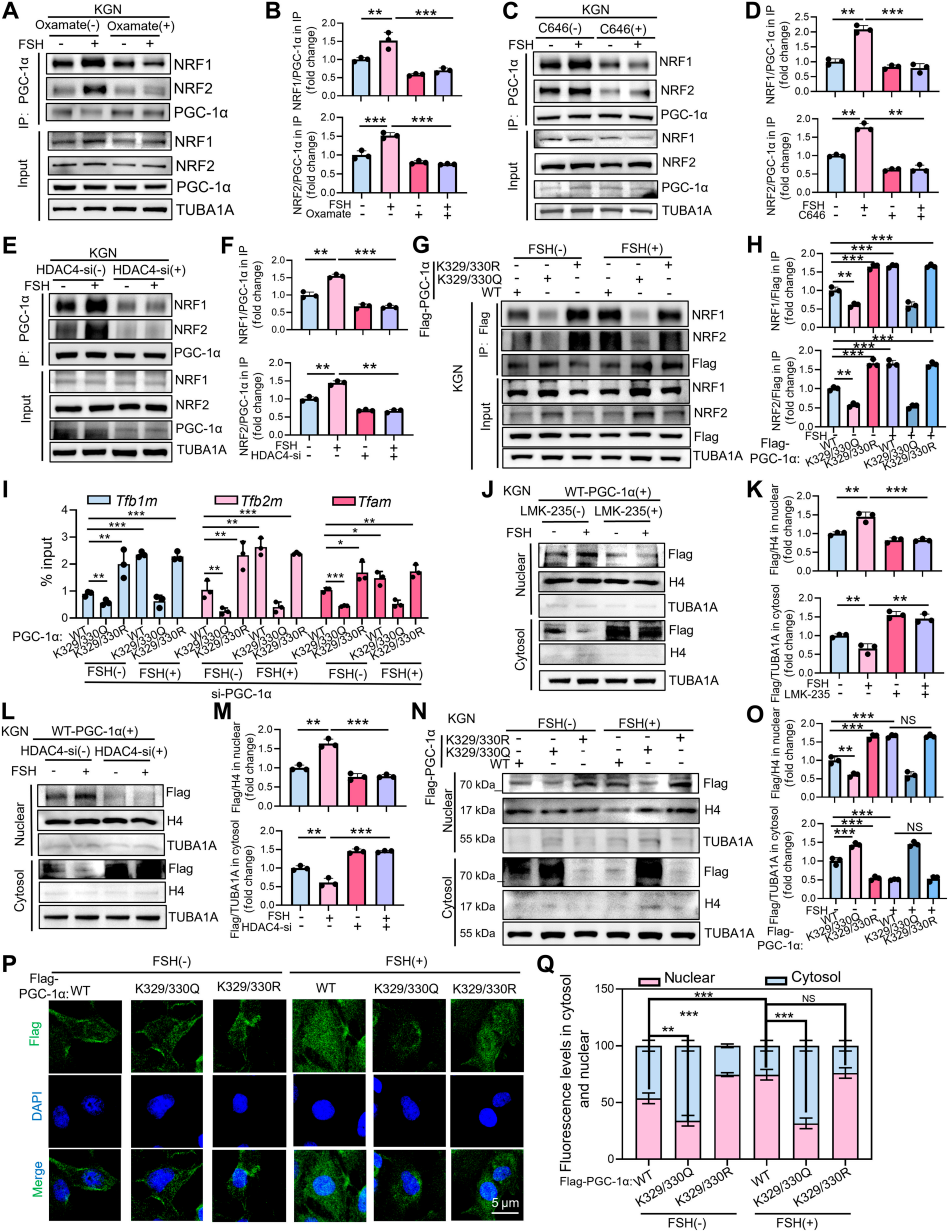
Deacetylation of PGC-1α enhances its interaction with NRF1/2. (A) Analysis of the interaction between PGC-1α and NRF1/2 by IP in KGN cells. Cells were first treated with 15 mM oxamate for 2 h, followed by stimulation with 5 IU of FSH for 12 h. (B) Quantitative measurement of the binding affinity between PGC-1α and proteins NRF1/NRF2 in (A). The affinity is presented as the level of NRF1 or NRF2 co-immunoprecipitated with PGC-1α, normalized to the total PGC-1α protein level. (C) Co-IP assays examining PGC-1α binding to NRF1/2 within KGN cells: samples pretreated with 10 μM C646 (2 h) and then stimulated with FSH (12 h). (D) Quantitative measurement of the binding affinity between PGC-1α and proteins NRF1/NRF2 in (C). The affinity is presented as the level of NRF1 or NRF2 co-immunoprecipitated with PGC-1α, normalized to the total PGC-1α protein level. (E) Co-IP assay assessing PGC-1α and NRF1/2 binding in KGN cells post-HDAC4 knockdown (12 h) and FSH exposure (5 IU, 12 h). (F) Quantitative measurement of the binding affinity between PGC-1α and proteins NRF1/NRF2 in (E). The affinity is presented as the level of NRF1 or NRF2 co-immunoprecipitated with PGC-1α, normalized to the total PGC-1α protein level. (G) Co-IP analysis of PGC-1α/NRF1/2 binding dynamics in KGN cells expressing Flag-tagged WT, K329/330R (acetylation-resistant), or K329/330Q (acetylation-mimic) PGC-1α, treated with or without 5 IU of FSH for 12 h. (H) Quantitative measurement of the binding affinity between PGC-1α and proteins NRF1/NRF2 in (G). The affinity is presented as the level of NRF1 or NRF2 co-immunoprecipitated with PGC-1α, normalized to the total PGC-1α protein level. (I) KGN cells were first transfected with PGC-1α siRNA for 12 h, followed by overexpression of Flag-tagged constructs: WT PGC-1α, acetylation-resistant K329/330R PGC-1α, or acetylation-mimetic K329/330Q PGC-1α for 12 h, and then treated with 5 IU of FSH for an additional 12 h. ChIP analysis of the binding of Flag-tagged PGC-1α to the promoters of *Tfb1m*, *Tfb2m*, and *Tfam.* (J) KGN cells overexpressing Flag-tagged WT PGC-1α plasmid for 12 h were sequentially treated with 15 μM LMK-235 (2 h) followed by 5 IU of FSH (12 h). Subcellular fractionation was then performed to obtain cytosolic and nuclear extracts, which were subjected to immunoblot analysis using antibodies against Flag (transgene expression), TUBA1A (cytosolic marker), and histone H4 (nuclear marker). (K) PGC-1α levels in the nuclear and cytoplasmic fractions were quantified in (J). H4 and TUBA1A were used as internal controls for normalizing the nuclear and cytoplasmic proteins, respectively. (L) Immunoblot analysis was performed to examine Flag-tagged WT PGC-1α expression and subcellular localization in HDAC4-knockdown KGN cells. After 12-h Flag-PGC-1α induction, cells received 5 IU of FSH for another 12 h, and cytosolic and nuclear fractions were probed for Flag, TUBA1A (cytosolic marker), and histone H3 (nuclear marker). (M) Quantitatively measure the subcellular distribution of PGC-1α between the nucleus and cytoplasm in (L). H4 and TUBA1A were used as internal controls for normalizing the nuclear and cytoplasmic proteins, respectively. (N) KGN cells were first transfected with PGC-1α siRNA for 12 h, followed by overexpression of Flag-tagged constructs: WT PGC-1α, acetylation-resistant K329/330R PGC-1α, or acetylation-mimetic K329/330Q PGC-1α for 12 h, and then treated with 5 IU of FSH for an additional 12 h. (O) Quantitatively measure the subcellular distribution of PGC-1α between the nucleus and cytoplasm in (N). H4 and TUBA1A were used as internal controls for normalizing the nuclear and cytoplasmic proteins, respectively. (P) Immunofluorescence analysis of PGC-1α subcellular localization in KGN cells transfected with Flag-tagged WT, K329/330R, or K329/330Q PGC-1α for 12 h, followed by treatment 5 IU of FSH for 12 h. (Q) Quantitative analysis of Flag fluorescence intensity from (P). Data are presented as the mean ± SD from at least 3 independent experiments (*n* ≥ 3). Statistical differences between groups were compared by one-way ANOVA followed by LSD post hoc test.

PGC-1α functions as a master regulator of mitochondrial biogenesis, cooperatively engaging transcription factors such as NRF1 and GABPA (NRF2) to activate nuclear genes required for mitochondrial function [19,35]. To determine how acetylation at K329/330 influences PGC-1α activity, we examined FSH-dependent changes in its association with transcriptional partners via co-IP. FSH stimulation markedly enhanced the interaction between PGC-1α and NRF1/NRF2 (Fig. 7A and B), whereas disruption of lactate-dependent H4K5la abolished this effect (Fig. 7A and B). Likewise, exposure to the P300/CBP inhibitor C646 or depletion of HDAC4 significantly attenuated FSH-induced PGC-1α–NRF1/2 complex formation in KGN cells (Fig. 7C to F). To assess the functional significance of K329/330 acetylation, we expressed Flag-tagged PGC-1α variants (WT, acetylation-deficient K329/330R, or acetylation-mimetic K329/330Q) in KGN cells. The K329/330R mutation enhanced PGC-1α binding to NRF1/NRF2 under FSH stimulation, whereas the K329/330Q variant substantially impaired these interactions (Fig. 7G and H). Consistent with these findings, ChIP-qPCR revealed increased promoter occupancy of NRF1/NRF2 target genes (*TFAM*, *TFB1M*, *TFB2M*) by the K329/330R mutant in FSH-treated cells, while the K329/330Q mutant displayed markedly reduced chromatin binding (Fig. 7I). Together, these results indicate that deacetylation of PGC-1α at K329/330 promotes its recruitment to NRF1/2 and drives activation of mitochondrial gene programs in response to FSH.

PGC-1α must translocate from the cytoplasm to the nucleus to function as a transcriptional coactivator. To determine whether acetylation regulates its subcellular distribution, we transfected KGN cells with Flag-tagged WT PGC-1α and performed nuclear–cytoplasmic fractionation. FSH treatment induced robust nuclear localization of PGC-1α, whereas inhibition of HDAC4 by LMK-235 or HDAC4-specific siRNA attenuated this nuclear accumulation (Fig. 7J to M). To further interrogate the role of K329/330 acetylation, we expressed WT, K329/330R, or K329/330Q PGC-1α constructs followed by subcellular fractionation. The K329/330R mutant enhanced FSH-stimulated nuclear entry of PGC-1α, whereas the K329/330Q mutant diminished nuclear localization (Fig. 7N and O). Immunofluorescence staining corroborated these results, demonstrating increased cytoplasm-to-nucleus translocation of the K329/330R variant and enhanced nuclear-to-cytoplasm redistribution of the K329/330Q mutant in FSH-treated cells (Fig. 7P and Q). These findings suggest that FSH reduces PGC-1α acetylation, promoting its nuclear entry and enhancing mitochondrial biogenesis.

### Inhibition of the H4K5la/HDAC4/PGC-1α pathway impairs FSH-induced mitochondrial biogenesis and follicular development in vivo

**Fig. 8.**
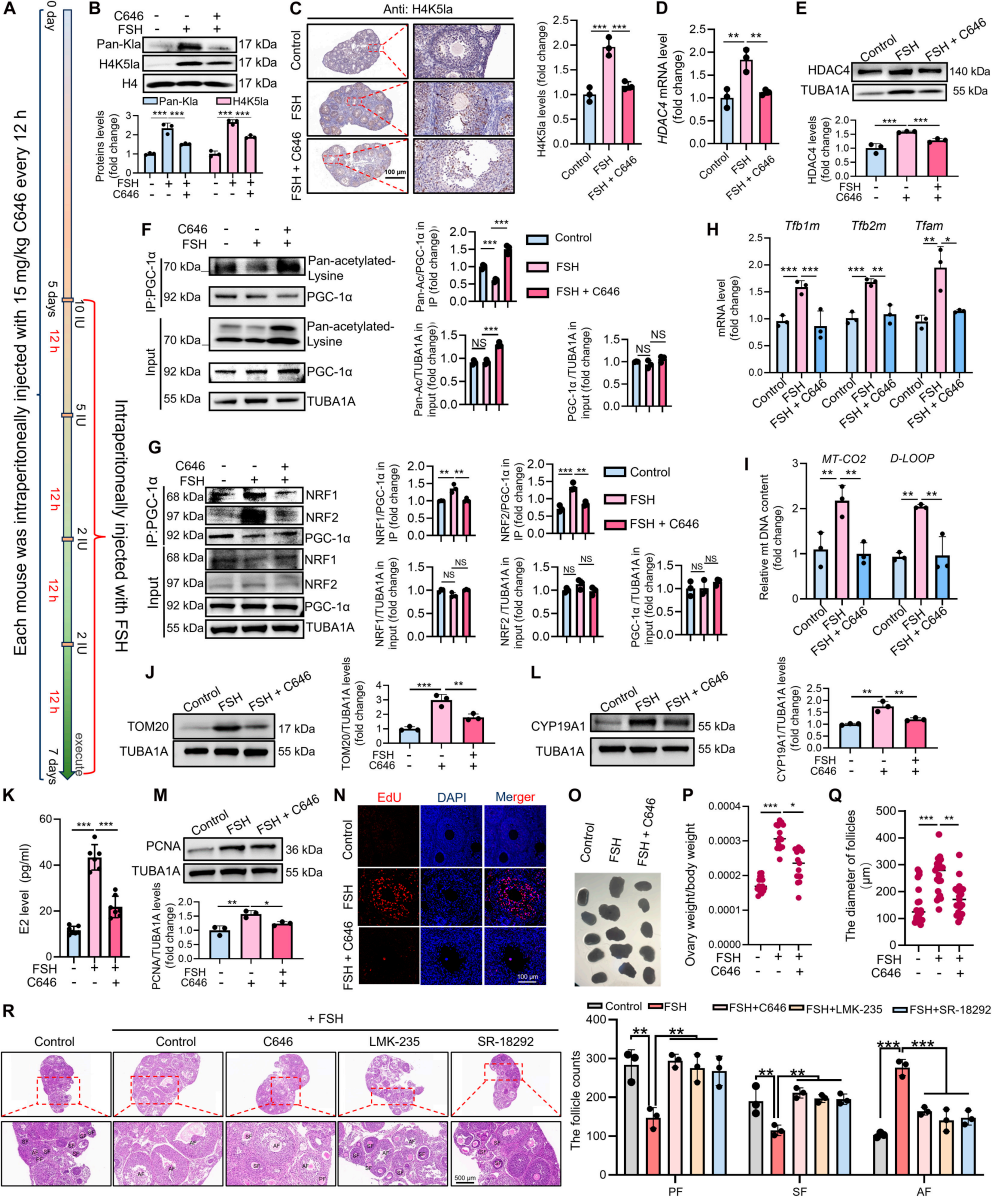
C646-mediated P300 inhibition inhibits mitochondrial biogenesis and follicular development in vivo. (A) Schematic diagram of the in vivo experimental procedure. Mice were randomly assigned to 5 groups: (1) control (DMSO/0.9% saline vehicle), (2) FSH alone, (3) FSH + C646 (15 mg/kg), (4) FSH + LMK-235 (15 mg/kg), and (5) FSH + SR-18292 (15 mg/kg). All intraperitoneal injections were administered at 12-h intervals. The FSH regimen followed a tapering protocol of 10 IU, 5 IU, and two 2-IU doses. The respective inhibitors were co-administered with each FSH injection. All drugs were dissolved in DMSO and diluted in 0.9% saline for administration. (B) Western blot analysis of Pan-Kla within histone regions and H4K5la levels following the indicated treatments in (A), with H4 used as a loading control for normalization. (C) Immunohistochemical detection of Pan-Kla expression following the indicated treatments in (A). Pan-Kla^+^ normalized to total cell number. Scale bar, 200 μm. (D) qRT-PCR measurement of *HDAC4* expression after specified treatments in (A). *Tuba1a* served as the loading control for data normalization. (E) Western blot assessment of HDAC4 expression posttreatment in (A), with TUBA1A used as a loading control for normalization. (F) Co-IP assay assessing PGC-1α and pan-acetyl-lysine binding posttreatment in (A). For IP, PGC-1α acetylation was quantified as the ratio of acetylated to total PGC-1α. For Input, the levels of total acetylation and PGC-1α protein were normalized to TUBA1A. (G) Co-IP assay assessing PGC-1α and NRF1/2 binding posttreatment in (A). For IP, the binding of PGC-1α to NRF1/2 was measured by calculating the NRF1/2 to PGC-1α ratio. For Input, the levels of NRF1/2 and PGC-1α were normalized to TUBA1A. (H) qRT-PCR examination of *Tfb1m*, *Tfb2m*, and *Tfam* mRNA expression after the specified treatments in (A). *Tuba1a* served as the loading control for data normalization. (I) qRT-PCR was used to assess mitochondrial DNA copy number, specifically targeting the *MT-CO2* and *D-Loop* regions, following the indicated treatments in (A). *β-Actin* served as the loading control for data normalization. (J) Western blot assessment of TOM20 expression posttreatment in (A), with TUBA1A used as a loading control for normalization. (K) Using a radioimmunoassay (RIA), we quantified the serum estradiol (E2) concentrations across the treatment groups specified in (A). (L) Western blot assessment of CYP19A1 expression posttreatment in (A), with TUBA1A used as a loading control for normalization. (M) Western blot assessment of proliferating cell nuclear antigen (PCNA) expression posttreatment in (A), with TUBA1A used as a loading control for normalization. (N) 5-Ethynyl-2’-deoxyuridine (EdU) incorporation assay detects the proliferation activity of mouse ovarian GCs following the indicated treatments in (A). EdU-positive cells normalized to total cell number. Scale bar, 100 μm. (O) Measurement of ovarian size following the indicated treatments in (A). (P) Measurement of ovarian weight following the indicated treatments in (A). The ovary weight was expressed relative to the body weight of the corresponding mouse. (Q) Measurement of follicle diameter following the indicated treatments in (A). (R) The counts of primary, secondary, and antral follicles were assessed via hematoxylin and eosin (H&E) staining as outlined in treatment (A). PF, primary follicle; SF, secondary follicle; AF, antral follicles. Scale bar, 500 μm. Data are presented as the mean ± SD from at least 3 independent experiments (*n* ≥ 3). Statistical differences between groups were compared by one-way ANOVA followed by LSD post hoc test.

To evaluate the physiological relevance of the H4K5la/HDAC4/PGC-1α pathway, mice receiving FSH were treated with inhibitors targeting key components of this axis: C646 (P300/CBP lactylation inhibitor), LMK-235 (HDAC4 antagonist), or SR-18292 (PGC-1α acetylation activator) (Fig. 8A and Fig. S8A). C646 treatment abolished FSH-induced H4K5la in ovarian GCs (Fig. 8B and C) and concomitantly suppressed HDAC4 expression (Fig. 8D and E). Consistent with HDAC4-mediated PGC-1α deacetylation, pathway inhibition increased PGC-1α acetylation (Fig. 8F and Fig. S8B and C) and disrupted PGC-1α–NRF1/NRF2 interactions (Fig. 8G and Fig. S8D and E).

Importantly, blockade of this pathway markedly impaired FSH-induced mitochondrial biogenesis, as reflected by reduced mitochondrial gene expression (Fig. 8H and Fig. S8F), decreased mtDNA copy number (Fig. 8I and Fig. S8G), and diminished TOM20 protein levels (Fig. 8J and Fig. S8H and I). Mitochondrial deficits were accompanied by reduced estradiol (E2) production (Fig. 8K and L and Fig. S8J to L) and decreased GC proliferation (Fig. 8M and N and Fig. S8M and N). At the organ level, inhibitor-treated mice exhibited smaller ovaries (Fig. 8O and Fig. S8O), lower ovarian weight (Fig. 8P and Fig. S8P), arrested follicular development (Fig. 8Q), and reduced formation of antral follicles (Fig. 8R). Collectively, these findings demonstrate that the H4K5la/HDAC4/PGC-1α axis is essential for FSH-mediated follicular development by supporting mitochondrial metabolic adaptation.

## Discussion

**Fig. 9.**
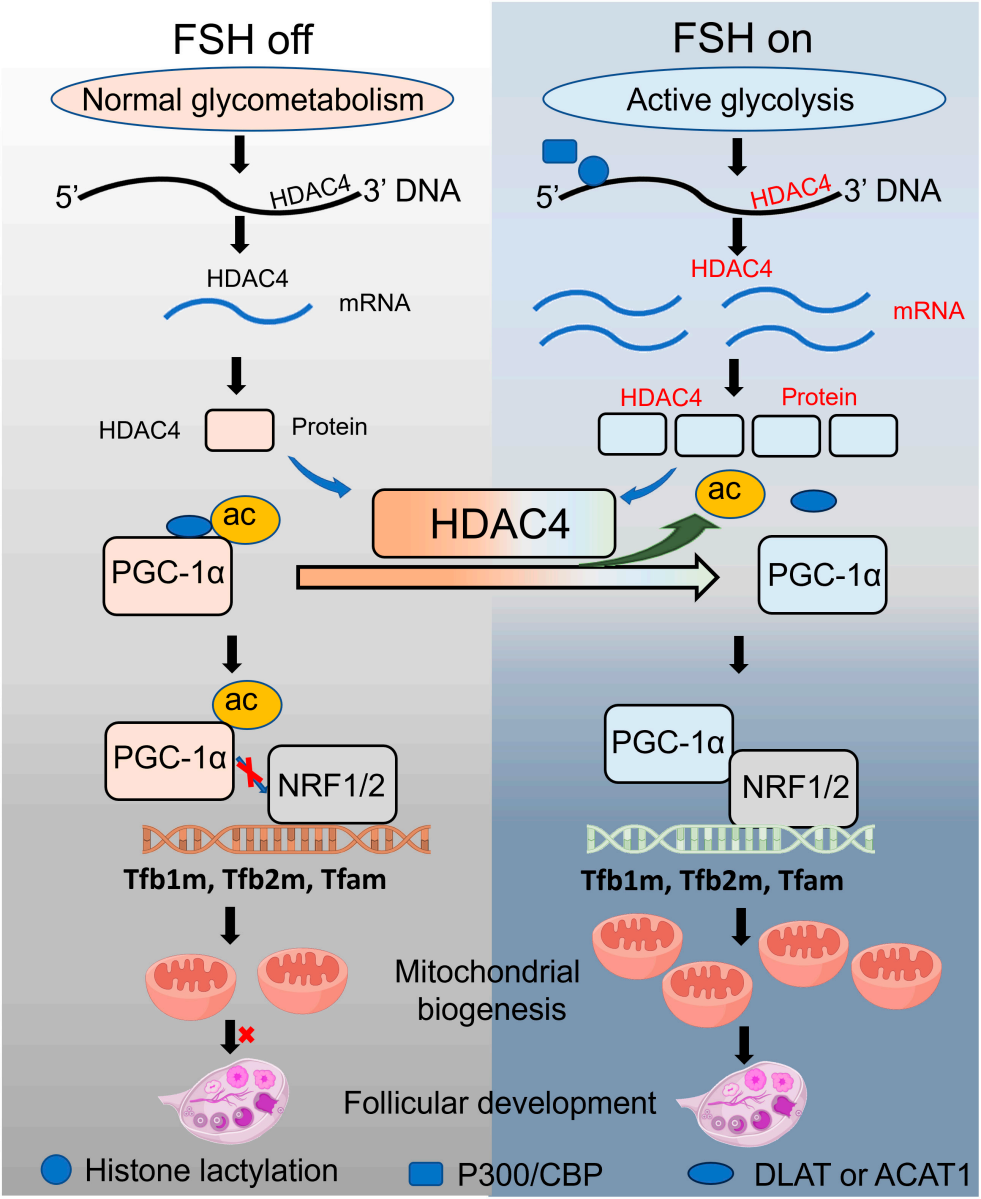
Mechanistic model of H4K5la in FSH-driven mitochondrial biogenesis. FSH activates aerobic glycolysis in GCs, generating lactate that induces histone H4K5la via P300/CBP. This epigenetic modification promotes HDAC4 transcription, leading to HDAC4-mediated deacetylation of PGC-1α at K329/330. Deacetylated PGC-1α facilitates the recruitment of nuclear respiratory factors (NRF1 and NRF2) to promoter regions, initiating the expression of essential genes involved in mitochondrial biogenesis, such as *TFAM*, *TFB1M*, and *TFB2M*. This process ultimately leads to an expansion of the mitochondrial network. Through this lactate–H4K5la–HDAC4 axis, FSH synchronizes mitochondrial expansion with the bioenergetic demands of follicular development.

The dynamic coordination between glycolytic and mitochondrial metabolism is crucial to sustain the energetic demands of follicular growth [36–38]. Although FSH-enhanced glycolysis and lactate production are established, our study reveals a previously unrecognized role for lactate as an epigenetic signal that directly licenses mitochondrial biogenesis through histone lysine lactylation. We show that FSH-induced H4K5la, mediated by P300/CBP, serves as a metabolic checkpoint to activate HDAC4 expression. The HDAC4–PGC-1α–NRF1/NRF2 cascade subsequently drives transcription of mitochondrial biogenesis genes, ensuring adequate energy generation to support the proliferative and steroidogenic demands of GCs during folliculogenesis (Fig. 9). More broadly, our findings reveal that lactate-derived histone lactylation functions as a rheostat for mitochondrial capacity, illustrating how metabolites dynamically shape chromatin to couple cellular energetics with developmental cues.

As the primary site of TCA cycle activity, mitochondria rely on pyruvate as a critical metabolic substrate bridging glycolysis and oxidative phosphorylation [39]. Following mitochondrial import, pyruvate undergoes pyruvate dehydrogenase (PDH)-mediated decarboxylation to acetyl-coenzyme A (CoA), fueling the TCA cycle [40–42]. Lactate, traditionally viewed as a glycolytic byproduct, can be converted to pyruvate by LDH and serves as an alternative substrate for oxidative metabolism [27]. Recent studies demonstrate that lactate enters mitochondria through MCT1 and undergoes NAD^+^ [nicotinamide adenine dinucleotide (oxidized form)]-dependent conversion to pyruvate by mitochondrial LDH, establishing lactate as a direct precursor for the TCA cycle [27]. Beyond this metabolic role, our findings uncover a novel mechanism whereby lactate regulates mitochondrial biogenesis through histone lactylation, providing a mechanistic link between glycolytic flux and epigenetic control of mitochondrial function.

Mitochondrial biogenesis is governed by multilayered epigenetic regulation, including histone acetylation (e.g., H4K12ac and H3K9ac) [43,44] and methylation (e.g., H3K79me3 at the SIRT3 promoter) [45], which coordinate expression of nuclear-encoded mitochondrial genes. Posttranslational modifications of nonhistone proteins further refine mitochondrial homeostasis. For example, KDM3A-mediated demethylation of PGC-1α at K224 reduces its transcriptional synergy with NRF1/2 [19], while GCN5L1-dependent acetylation of TFAM at K76 impairs mitochondrial protein import [46]. These findings highlight intricate crosstalk between chromatin state and mitochondrial regulation. Our study expands this framework by identifying histone lactylation as a novel epigenetic layer. Specifically, FSH-induced H4K5la activates HDAC4 to deacetylate PGC-1α and promote NRF1/NRF2-dependent transcriptional programs while also complementing nonhistone acetylation events to support mitochondrial proteostasis. Thus, histone lactylation emerges as a central integrator coordinating multiple posttranslational modifications to fine-tune mitochondrial function.

Although lactylation modifies both histone and nonhistone proteins [47–52], the distinct functional consequences of nuclear versus cytoplasmic localization remain incompletely understood. Here, we demonstrate that FSH-induced lactylation is predominantly enriched within the nucleus of GCs, with histone H4K5la identified by MS/MS as a key regulator of mitochondrial biogenesis. This spatial specificity suggests that nuclear histone lactylation, rather than cytoplasmic nonhistone lactylation, serves as the primary epigenetic driver of mitochondrial adaptation. Nevertheless, a cooperative role for nuclear nonhistone lactylation cannot be excluded, as it may fine-tune mitochondrial dynamics by modulating chromatin-associated enzymes or transcriptional cofactors. Histone and nonhistone lactylation likely act through complementary but mechanistically distinct pathways: Histone lactylation primarily governs mitochondrial biogenesis at the transcriptional level, whereas nonhistone lactylation may modulate mitochondrial proteostasis and metabolic activity. Future studies using spatial proteomics and subcellular lactylome profiling will be required to dissect their interplay. In addition, lactylation-deficient knock-in models (e.g., H4K5R) will help clarify the hierarchical contributions of these modifications to follicular development and reproductive competence.

Previous studies have demonstrated that PGC-1α is a direct substrate of HDAC4 and that HDAC4-mediated deacetylation occurs at lysine residues K329/330 on PGC-1α [24]. However, the functional importance of this deacetylation in mitochondrial biogenesis had not been established. Our findings identify a previously unrecognized pathway in which PGC-1α deacetylation at K329/330 enhances its binding to NRF1/2, thereby coordinating the mitochondrial biogenesis program. Moreover, we show that histone H4K5la specifically targets HDAC4, facilitating PGC-1α deacetylation and subsequently promoting NRF1/2 recruitment and transcription of mitochondrial biogenesis genes. Thus, we uncover a mechanism whereby HDAC4-dependent PGC-1α deacetylation, regulated by H4K5la, drives mitochondrial biogenesis to support GC proliferation and differentiation, ultimately promoting follicular development.

Our findings reveal a novel regulatory axis in which HDAC4-mediated PGC-1α deacetylation, modulated by histone H4K5la, stimulates GC growth and specialization, thereby accelerating ovarian follicle maturation. This work provides new insight into the molecular interplay governing reproductive cell development and advances our understanding of the mechanisms underlying FSH-induced mitochondrial biogenesis and follicular progression.

P300 functions as a dual writer for both lactylation and acetylation [53–56]. In our study, FSH markedly increased global lactylation without detectable changes in global acetylation. Pharmacological inhibition of P300 abolished lactylation but did not affect overall acetylation, suggesting compensatory mechanisms that maintain acetylation under FSH stimulation. Notably, P300 inhibition also suppressed FSH-induced HDAC4 expression, indicating that P300 mediates HDAC4 transcription downstream of FSH and thereby indirectly influences the acetylation landscape. Consistent with this model, FSH reduced PGC-1α acetylation, a change more readily explained by enhanced HDAC4 activity than by altered P300-catalyzed acetylation. Together, these observations support the existence of a dynamic, metabolically responsive “acetylation–lactylation” balance in which FSH elevates lactate and histone lactylation via P300 while simultaneously activating HDAC4 to remodel acetylation states. Future studies should assess competition at shared lysine residues, determine whether lactylation and acetylation are mutually exclusive at specific sites, and identify reader proteins that interpret these marks to build an integrated posttranslational regulatory network.

Similarly, inhibition or knockdown of P300/CBP activity or expression markedly suppressed FSH-induced Pan-Kla, whereas the decrease in lactylation at H4K5 was comparatively modest. This differential effect may result from P300 inhibition, causing a pronounced loss of lactylation at sites fully dependent on its enzymatic function, thereby substantially reducing the Pan-Kla signal. By contrast, H4K5, as a critical chromatin modification site, is likely regulated through additional finely tuned mechanisms. In this context, P300/CBP depletion may indirectly modulate other regulatory factors involved in H4K5la deposition, partially buffering the decline in lactylation at this site. Nonetheless, our data clearly demonstrate that P300/CBP functions as the writer enzyme for H4K5la and is essential for FSH-induced mitochondrial biogenesis in GCs. Taken together, the distinct responses of Pan-Kla and H4K5la to P300 inhibition support the notion that P300 serves as a central driver of global protein lactylation under FSH stimulation, whereas regulation of the specific H4K5 locus is integrated within this broader network and subjected to additional hierarchical control. Future studies will aim to dissect these regulatory layers in detail. Importantly, these observations do not weaken our conclusions; rather, they underscore the pivotal role of P300 in lactylation biology and reveal the nuanced complexity of its regulatory landscape.

Our study primarily evaluated the signaling function of lactate, and its dual role in the follicular microenvironment requires further investigation. As a metabolic substrate, lactate enters GCs via monocarboxylate transporters and is converted to pyruvate by LDH to supply the TCA cycle, supporting ATP production and follicular growth. Concurrently, lactate acts as a signaling molecule that modulates gene-regulatory networks through histone lactylation. The relative balance between these metabolic and signaling functions is likely shaped by oxygen gradients, intercellular metabolic coupling, and transporter expression within the follicle, forming a microenvironmental regulatory framework essential for normal folliculogenesis.

Although our in vitro data demonstrate that PGC-1α K329/330R and K329/330Q mutants differentially regulate TFAM, TFB1M, and TFB2M expression in FSH-stimulated GCs, the physiological relevance of these acetylation-site mutations remains to be established. Future in vivo studies employing knock-in models carrying PGC-1α acetylation-site mutations will be crucial to define their role in mitochondrial biogenesis and folliculogenesis. Furthermore, while we confirmed that FSH-induced H4K5la regulates mitochondrial biogenesis, the upstream mechanisms governing its precise regulation remain unclear. Future work should focus on identifying the writers, erasers, and readers of H4K5la to delineate this posttranslational modification pathway. Finally, whether lactate regulates mitochondrial biogenesis through similar mechanisms in other cell types warrants further investigation.

## Conclusion

This study delineates a lactate–H4K5la–HDAC4 axis that couples FSH-stimulated glycolysis to mitochondrial biogenesis. Mechanistically, FSH-induced histone H4K5la activates HDAC4 transcription, leading to PGC-1α deacetylation and enhanced interaction with NRF1/2, thereby promoting mitochondrial biogenesis to satisfy the metabolic demands of follicular development. These findings redefine lactate as a dual-function metabolite that fuels mitochondrial respiration while serving as an epigenetic substrate. By converting glycolytic flux into chromatin-modifying signals via H4K5la, histone lactylation emerges as a dynamic rheostat that synchronizes mitochondrial capacity with hormonal cues. This work advances our understanding of reproductive energy metabolism and highlights potential therapeutic opportunities for metabolic dysfunction in reproductive disorders.

## Materials and Methods

### Animals

All animal experiments were performed in compliance with the ethical standards established by Nanjing Agricultural University’s Institutional Animal Care and Use Committee, following internationally recognized guidelines for laboratory animal welfare (Nanjing, China). Three- to 4-week-old female ICR mice, procured from Qing Long Shan Co. Animal Breeding Center (Nanjing, China), were placed in a thermostatically controlled environment (the temperature was maintained at 22 ± 2 °C and a 12-h day and night cycle) with adequate food and water supply. They lived in groups, sharing a room for every 5 animals. The animals were randomly divided into 5 treatment arms: (a) saline-injected controls, (b) FSH only, (c) FSH plus C646, (d) FSH plus LMK-235, and (e) FSH plus SR-18292. The FSH regimen consisted of intraperitoneal doses (10, 5, 2, and 2 IU, in saline) administered at 12-h intervals. Cotreatment groups received identical FSH injections combined with either 15 mg/kg C646, 20 mg/kg LMK-235, or 45 mg/kg SR-18292. All compounds were prepared in a dimethyl sulfoxide (DMSO)/0.9% saline solution and delivered intraperitoneally. Control mice were injected with the vehicle solution at a volume matching that of the treatment groups. Following 7 d of intervention, all subjects were humanely euthanized, and GCs were collected for subsequent evaluation.

### Chemicals and immunological reagents

The following reagents were sourced commercially for this study: 2-DG (S4701), sodium oxamate (S6871), C646 (S7152), and LMK-235 (S7569) from Selleck Chemicals; FSH originates from Ningbo Second Hormone Factory (Zhejiang, China); PTM Bio provides an anti-l-lactylysine antibody (PTM-1401RM) that targets modified lysine residues, and a lactylylated histone H4 (K5) antibody (PTM-1407RM) that specifically recognizes the lactonated modification of the fifth lysine of histone H4. Additional antibodies included P300 (86377S), CBP (7389S), PCNA (2586S), TOM20 (42406S), PGC-1α (2178S), and Flag (8146S) from Cell Signaling Technology; histone H4 (16047-1-AP), HDAC4 (66838-1-AP), NRF1 (66832-1-AP), GLUT1 (21829-1-AP), TUBA1A (11224-1-AP), ACAT1 (16215-1-AP), DLAT1 (13426-1-AP), and LDHA (21799-1-AP) from Proteintech; LDHB (PAB698Hu01) from Cloud-Clone Corp.; and NRF2 (PA5-27735) from Thermo Fisher Scientific.

### Cell culture and drug treatment

GCs were isolated from 3- to 4-week-old juvenile ICR female mice using a standardized protocol. Mice were administered 10 IU of pregnant mare serum gonadotropin (PMSG) and euthanized 48 h post-injection. Ovarian tissues were collected, and mouse GCs (mGCs) were harvested by follicular aspiration using a 26-gauge needle connected to a 1-ml syringe. The isolated mGCs were cultured in Dulbecco’s modified Eagle’s medium (DMEM)/F-12 medium supplemented with 10% heat-inactivated fetal bovine serum and 1% penicillin–streptomycin, and maintained at 37 °C in a humidified atmosphere of 5% CO₂.

The human granulosa-like tumor cell line KGN (YILI Biology, Shanghai) was cultured under identical conditions. For pharmacological interventions, both mGCs and KGN cells were pretreated for 2 h with either metabolic inhibitors (15 mM 2-DG or 15 mM oxamate) or signaling modulators (10 μM C646 or 15 μM LMK-235), followed by stimulation with 10 IU/ml FSH for 12 h. The compounds were prepared as follows: 2-DG and oxamate were dissolved in ultrapure water and applied at 15 mM, with control groups receiving an equal volume of ultrapure water; C646 and LMK-235 were dissolved in DMSO and administered at 10 and 15 μM, respectively, with corresponding controls treated with an equivalent volume of DMSO. All experiments included triplicate biological replicates to ensure reproducibility.

### ^125^I-labeled E2 radioimmunoassay

Serum E2 levels were quantified through a competitive binding radioimmunoassay (RIA) system (Beijing North Biotechnology Research Institute, China). The testing was conducted in adherence to the manufacturer’s established protocols. The assay principle involves competitive binding between endogenous E2 and ^125^I-labeled E2 (^125^I-E2) for limited antibody binding sites. The assay procedure involved mixing test samples and calibration standards with iodine-125-labeled E2 (^125^I-E2) tracer solution and anti-E2 polyclonal antibodies in dedicated reaction vessels. This mixture was subjected to a 90-min incubation period at physiological temperature (37 °C) to facilitate competitive immunoreaction between native and radiolabeled E2 for antibody binding sites. Following incubation, an immunoseparation reagent (PR) was added to separate bound and free fractions, with subsequent centrifugation at 3,600*g* for 20 min. Quantification of antibody-bound complexes was performed through gamma radiation detection. A calibration curve correlated known E2 concentrations with observed bound radioactivity percentages, enabling sample E2 levels to be interpolated from their respective binding percentages.

### Western blotting

Proteins from cells were isolated with the radioimmunoprecipitation assay (RIPA) lysis buffer (ST506, Beyotime Biotechnology), supplemented with 1 mM phenylmethylsulfonyl fluoride (PMSF) for protease control. Protein quantification was performed with a bicinchoninic acid (BCA) assay system (Beyotime Biotechnology, P0012S), with absorbance measured at 562 nm. For Western blot analysis, protein samples (30 μg per lane) were separated on 10% SDS-PAGE (polyacrylamide gel electrophoresis) gels and subsequently transferred to polyvinylidene difluoride (PVDF) membranes (Millipore, 0.45 μm pore size) via semi-dry electrophoretic transfer. To prevent nonspecific binding, the membranes were incubated for 2 h at 25 °C in a tris-buffered saline solution with 0.1% Tween 20, all diluted to 5% bovine serum albumin (BSA). Subsequently, they were probed with primary antibodies diluted at a ratio of 1:1,000 in the blocking solution, and this process was carried out under refrigeration overnight. After triplicate 10-min TBST (tris-buffered saline with Tween 20) rinses, membranes underwent a 2-h room temperature incubation with horseradish peroxidase (HRP)-linked secondary antibodies (1:2,000 dilution, Abcam). Signal detection was achieved using an enhanced chemiluminescence substrate, with band intensity quantification performed using ImageJ software (National Institutes of Health). Expression data for each protein were adjusted relative to α-tubulin (TUBA1A) for normalization.

### Quantitative real-time PCR

RNA from cells was extracted using TRIzol (Invitrogen) via standard phenol–chloroform methods. To kick off the process, we synthesized first-strand cDNA from 1 μg of total RNA using Takara Bio’s PrimeScript RT Master Mix, all in a 20-μl reaction. Jumping into qPCR, we leveraged SYBR Green chemistry (Vazyme) on an Applied Biosystems StepOne Plus thermal cycler. The cycling conditions were as follows: an initial denaturation step at 95 °C for 30 s, followed by 40 cycles of denaturation at 95 °C for 5 s and annealing/extension at 60 °C for 30 s. Gene expression analysis employed sequence-specific primers (detailed in Table S1). Every test was run in technical triplicate, ensuring that negative controls were in place. For relative quantification, the 2^−ΔΔ*C*t^ formula was applied, and results were normalized using the endogenous reference gene Tuba1a.

### Immunofluorescence staining

For subcellular localization studies, the KGN cells grown on glass slides were first treated with a fresh 4% solution of paraformaldehyde (PFA) diluted in phosphate-buffered saline (PBS), left to fix for a solid 30 min at room temperature. Cellular membranes were permeabilized using 0.05% Triton X-100 (v/v) in PBS for 10 min at 4 °C, followed by blocking with 5% BSA (w/v) in PBS for 2 h at room temperature to prevent nonspecific binding. The primary antibodies were incubated overnight at 4 °C in a humidity-controlled chamber, diluted in PBS supplemented with 1% BSA. Following thorough PBS washes, the samples were treated with Alexa Fluor-tagged secondary antibodies (diluted 1:500 in 1% BSA–PBS) for 2 h, shielded from light to prevent photobleaching. Nuclear staining was performed using DAPI (4′,6-diamidino-2-phenylindole; 1 μg/ml) for 10 min at ambient temperature. The images were acquired with a Zeiss LSM 900 Airyscan confocal microscope, where excitation wavelengths and photomultiplier tube gain parameters were individually calibrated for each fluorescent probe to ensure optimal signal-to-noise ratio.

### RNA isolation, library assembly, and nucleotide analysis

RNA extraction from mGCs was conducted using the TRIzol reagent from Invitrogen. For each RNA sample, strand-specific cDNA libraries were constructed following Illumina’s TruSeq Stranded mRNA Library Prep Kit protocol (San Diego, CA), adhering closely to the manufacturer’s guidelines. Illumina HiSeq X (Illumina, San Diego, CA) facilitated extensive sequencing, yielding initial datasets. Quality control processing involved the removal of adapter contaminants and low-quality sequences, which was then followed by aligning the polished reads with the mouse reference genome [GRCm39, National Center for Biotechnology Information (NCBI)] using Hisat2 software. Transcript abundance was estimated as FPKM (fragments per kilobase of transcript per million mapped reads) values through Cufflinks analysis. An analysis of differential gene expression was carried out using DESeq2, with adjusted *P* values determining statistical relevance. Functional annotations for genes that showed variation were then evaluated through Gene Ontology (GO) term enrichment analysis, employing Fisher’s exact test to assess significance.

### CUT&Tag experimental methodology

CUT&Tag libraries were constructed using the Hyperactive In-Situ ChIP Library Prep Kit for Illumina (catalog no. TD903-TD904, Vazyme Biotech Co. Ltd.) following the manufacturer’s instructions. Briefly, cells were immobilized using concanavalin A-coated magnetic beads and permeabilized with digitonin. The bead-bound cells were then sequentially incubated with a primary antibody targeting (K4K5la), a corresponding secondary antibody, and the Hyperactive pA/pG-Tn5 Transposon. This procedure enables the precise recruitment of Tn5 transposase to the genomic DNA regions bound by the target protein. The tethered Tn5 transposase simultaneously cleaves DNA and ligates adapter sequences (P5 and P7) to the fragmented ends. The resulting DNA fragments were then amplified by PCR using primers harboring the P5 and P7 adaptors to construct the sequencing library. The purified libraries were assessed for quality using an Agilent 2100 Bioanalyzer. Libraries passing quality control were sequenced on an Illumina NovaSeq 6000 platform to generate 150 base pairs (bp) paired-end reads for subsequent analysis.

Raw sequencing data were processed with fastp software to remove adapters and filter out low-quality reads, yielding high-quality clean reads. These clean reads were then aligned to the mouse reference genome GRCm39 using Bowtie2. Protein–DNA interaction peaks (peaks) were identified using SEACR under the “stringent” mode. Genomic annotation of the identified peaks was performed using ChIPseeker to determine their associated genes and genomic features. To decipher the sequence preferences of the binding sites, de novo motif discovery was conducted within the peak regions using MEME and DREME. The identified motifs were compared and annotated against known databases using Tomtom. Differential binding analysis was performed using the DiffBind R package. This process involved integrating peak sets from replicate samples, calculating read counts for consensus peaks, and identifying statistically significant differential binding sites based on binding affinity. Peaks with *P* < 0.05 and absolute log_2_ (fold change) > 1 were defined as statistically significant differential binding sites in this study.

### ChIP assay

The procedure for ChIP followed the standard operating procedure outlined in the Pierce Agarose ChIP Kit (Thermo Fisher Scientific, catalog no. 26156). In brief, cells were cross-linked with 1% formaldehyde to fix protein–DNA interactions, and the reaction was quenched by adding glycine. After washing with PBS, the cells were resuspended in lysis buffer containing protease inhibitors. The chromatin was then digested with micrococcal nuclease (MNase) at 37 °C to generate fragments consisting primarily of mononucleosomes (~150 to 200 bp). An aliquot of the digest was saved as the “Input” control. The remaining chromatin was incubated overnight at 4 °C with magnetic beads precoated with a specific antibody against the target protein (anti-H4K5la and anti-PGC-1α) or a control normal immunoglobulin G (IgG). The immunocomplexes were sequentially washed with low-salt and high-salt buffers and subsequently eluted using an elution buffer containing 0.1 M NaHCO₃ and 1% SDS. Finally, cross-links were reversed by incubation at 65 °C overnight, and proteins were digested by proteinase K. The DNA was purified via phenol–chloroform extraction and ethanol precipitation.

The purified ChIP-DNA and Input-DNA were used as templates for amplification with a SYBR Green master mix on a RT-PCR system. The amplification protocol was as follows: initial denaturation at 95 °C for 5 min; followed by 40 cycles of denaturation at 95 °C for 15 s and annealing/extension at 60 °C for 30 s. The specificity of the PCR products was verified by melt curve analysis. The enrichment of specific DNA fragments in the promoter regions of target genes was quantified using the 2^−ΔΔ*C*t^ method with gene-specific primers, normalized to the corresponding Input-DNA. Primer details can be found in Table S2

### Co-IP assay

For co-IP, cells were lysed in ice-cold IP lysis buffer (Beyotime) containing PMSF (Beyotime) and a protease inhibitor cocktail (Roche, 04693132001). The whole-cell lysates were then left to marinate overnight at a chill of 4 °C, in the company of antibodies that zero in on the target. Once the primary antibodies had made their mark, we tossed in 25 μl of protein A/G magnet beads and let the concoction simmer for an extra 2 h at 4 °C. Immunoprecipitated complexes, following thorough washing to eliminate nonspecific bindings, were analyzed using a Western blot technique with corresponding antibodies.

### Protein interaction profiling and acetylation enzyme screening

Protein complexes interacting with PGC-1α were isolated through IP with PGC-1α-specific antibodies, and their molecular constituents were subsequently analyzed via high-performance liquid chromatography (HPLC)–MS/MS. To elucidate potential modulators of PGC-1α acetylation, the acquired proteomic data were computationally integrated with known acylation-related enzymes using bioinformatic approaches.

### Quantification of lactate levels

The lactate concentration in both KGN and mGC cell cultures was quantified using a commercially available assay kit (A019-2-1, Nanjing Jiancheng Bioengineering Institute), following the protocol provided by the manufacturer. Absorbance readings of the chromogenic reaction products were taken at 570 nm with a TECAN microplate reader for spectrophotometric analysis. To account for potential variations in cell number, measured lactate values were standardized against total protein concentration as assessed by BCA protein quantification. Each experimental condition was analyzed in 3 independent replicates to maintain statistical reliability and experimental consistency.

### ATP assay

ATP levels were quantified with a commercial assay kit (BC0305, Solarbio), following the supplier’s instructions. For follicular fluid samples, mice were administered FSH as per the experimental design and subsequently sacrificed for ovary isolation, and follicular fluid was then aspirated via syringe puncture for ATP assay. For in vitro cultured cells, following the indicated drug treatments, cells were harvested by trypsinization and centrifugation and then subjected to ATP measurement, with all data normalized to the total protein concentration.

### Quantification of mitochondrial DNA

The extraction of genomic DNA was carried out using the QIAamp DNA Mini Kit (QIAGEN, Germantown), following the manufacturer’s recommended protocol. To assess relative mitochondrial DNA levels, we conducted qRT-PCR, targeting the mitochondrial D-Loop region and MT-CO2 gene expression, both normalized against the nuclear β-actin reference gene for accuracy. The specific primer sequences employed in this study are provided in Table S3.

### Immunohistochemical staining analysis

Ovarian specimens were preserved in 4% PFA, processed through paraffin embedding, and subjected to sequential alcohol dehydration (5% to 100% gradient). Thin tissue sections, cut to 5 μm with a microtome, were mounted onto glass slides. After removing the paraffin and rehydrating the samples, antigens were retrieved by heating the slides in citrate buffer using a microwave for half an hour. Endogenous peroxidases were inactivated by 10-min H_2_O_2_ exposure. Sections were then treated with 1% BSA blocking solution prior to overnight incubation with Pan-Kla, H4K5la, and Ki67 primary antibodies (1:200 dilution), followed by biotin-conjugated secondary antibody application. Chromogenic detection was performed using diaminobenzidine (DAB) substrate, with hematoxylin counterstaining applied before final dehydration. Digital imaging was conducted using an Olympus virtual microscopy system.

### Histological analysis and follicular quantification

For histological analysis, ovary specimens were subjected to 24-h fixation with a 4% PFA solution, followed by sequential dehydration in an ascending ethanol gradient and subsequent paraffin embedding. Thin tissue sections of 5-μm thickness were systematically obtained using a precision rotary microtome. For morphological evaluation and follicular enumeration, sections were subjected to standard eosin staining. Every fifth consecutive section was systematically examined to ensure comprehensive coverage while minimizing duplicate counts.

### RNA interference

The gene-specific siRNAs targeting P300, CBP, LDHA, HDAC4, PGC-1α, and LDHB along with nontargeting control siRNAs were purchased from GenePharma (Shanghai, China), while siRNAs against NAT10, DLAT, SCP2, ACAA1B, ACAT1, PAFAH1B3, FASN, ACAT3, LPCAT3, HADHB, PRDX6, HADHA, ACAA2, and AARS1 were obtained from GeneRay. The complete oligonucleotide sequences can be found in Table S4. For gene silencing experiments, siRNA delivery was performed employing Lipofectamine 3000 transfection agent (from Invitrogen), adhering to the manufacturer’s suggested procedure.

### MitoTracker Green staining

Following experimental treatments, KGN and mGC cells grown on coverslips were stained with 20 nM tetramethylrhodamine methyl ester (TMRM) and 50 nM MitoTracker Green FM at 37 °C for 30 min. Fluorescent signals from the cells were captured using a Zeiss LSM 710 confocal microscope setup. The resulting images were then analyzed for fluorescence intensity measurements with ImageJ (version 1.42q) to quantify the data.

### TEM for mitochondrial ultrastructure analysis

Following the FSH stimulation protocol, murine GCs were isolated by peritoneal puncture using a 1-ml syringe. Cell samples underwent initial fixation with 2.5% glutaraldehyde (Sigma-Aldrich, 49626), followed by postfixation in 1% osmium tetroxide (Sigma-Aldrich, 75632). After thorough washing, samples underwent graded ethanol dehydration before embedding in Araldite resin (Sigma-Aldrich, A3183). Ultrathin sections (50 nm thickness) were prepared, transferred to Formvar-coated copper grids, and stained with uranyl acetate (Polysciences, 6159-44-0). Morphological analysis was performed using a Hitachi H-7800 TEM (Hitachi, Tokyo, Japan), with mitophagic events identified by the presence of mitochondria within double-membrane autophagosomal structures. Quantitative assessment involved counting mitophagic vacuoles across 3 randomly selected cellular profiles per sample.

### JC-1 staining

The mitochondrial membrane potential was evaluated with the JC-1 assay kit (catalog no. 40706ES60), following the manufacturer’s established guidelines. Briefly, KGN cells were seeded in confocal-friendly dishes and exposed to experimental conditions. After treatment, the cells were incubated with a freshly prepared JC-1 working solution for 30 min at 37 °C, shielded from light. Dual-emission fluorescence signals were captured by confocal microscopy, with red fluorescence (J-aggregates, ~590 nm) indicating intact membrane potential and green fluorescence (monomeric form, 529 nm) reflecting depolarization. The red/green fluorescence quotient quantitatively indexed mitochondrial membrane potential.

### OCR and ECAR analysis

Mitochondrial respiration and glycolytic flux in KGN cells were measured concurrently via the Seahorse XF96 Extracellular Flux Analyzer. Experimentally, cells were adhered to 96-well assay plates at densities of 4 × 10^4^ or 8 × 10^4^ cells per well using Cell-Tak coating reagent (Corning). After equilibration in XF RPMI substrate-enriched medium (phenol red-free, with 10 mM glucose, 2 mM glutamine, and 1 mM sodium pyruvate) for 1 h, mitochondrial function was interrogated through sequential injections of 180 μl of assay medium, 20 μl of 2 μM oligomycin, 2 μM carbonyl cyanide-4-(trifluoromethoxy) phenylhydrazone, and 0.5 μM rotenone/antimycin A cocktail via the Cell Mito Stress Test Kit (Agilent) to determine OCR. Parallel glycolytic capacity assessment was performed using the Glycolysis Stress Test Kit (Agilent) with automated delivery of 10 mM glucose, 2 μM oligomycin, and 100 mM 2-DG for ECAR measurement. All metabolic parameters were normalized to cell counts for comparative analysis.

### Statistical analysis

The statistical analysis was conducted using GraphPad Prism (version 7.0), with all numerical data presented as mean ± standard deviation. Each experiment was replicated a minimum of 3 times under independent conditions. For group comparisons, a one-way analysis of variance (ANOVA) was applied, followed by the least significant difference (LSD) post hoc test to evaluate specific pairwise differences. A *P* value of less than 0.05 was considered statistically significant.

## Ethical Approval

All animal experiments were performed following the Nanjing Agricultural University Animal Care Committee guidelines (Nanjing, China) and in compliance with established international standards for laboratory animal welfare.

## Data Availability

The RNA-seq and CUT&Tag datasets generated in this study are publicly available in the NCBI database under accession codes PRJNA1209519 (https://www.ncbi.nlm.nih.gov/sra/?term=PRJNA1209519) and PRJNA1209605 (https://www.ncbi.nlm.nih.gov/sra/?term=PRJNA1209605), respectively. Additional supporting data are included in the manuscript and Supplementary Materials, with further information available upon request.
