# Supplementary material for: Histone Lactylation Couples FSH-Driven Lactate Metabolism to Mitochondrial Biogenesis by Enhancing HDAC4-Mediated Deacetylation of PGC-1α in Granulosa Cells
Source: Research (Wash D C). 2026 Jan 15;9:1045. doi: 10.34133/research.1045 (PMC12804605; doi:10.34133/research.1045)
Supplement: Supplementary 1 — Figs. S1 to S8 Tables S1 to S4 [file research.1045.f1.docx]

**Supplementary Information**

**
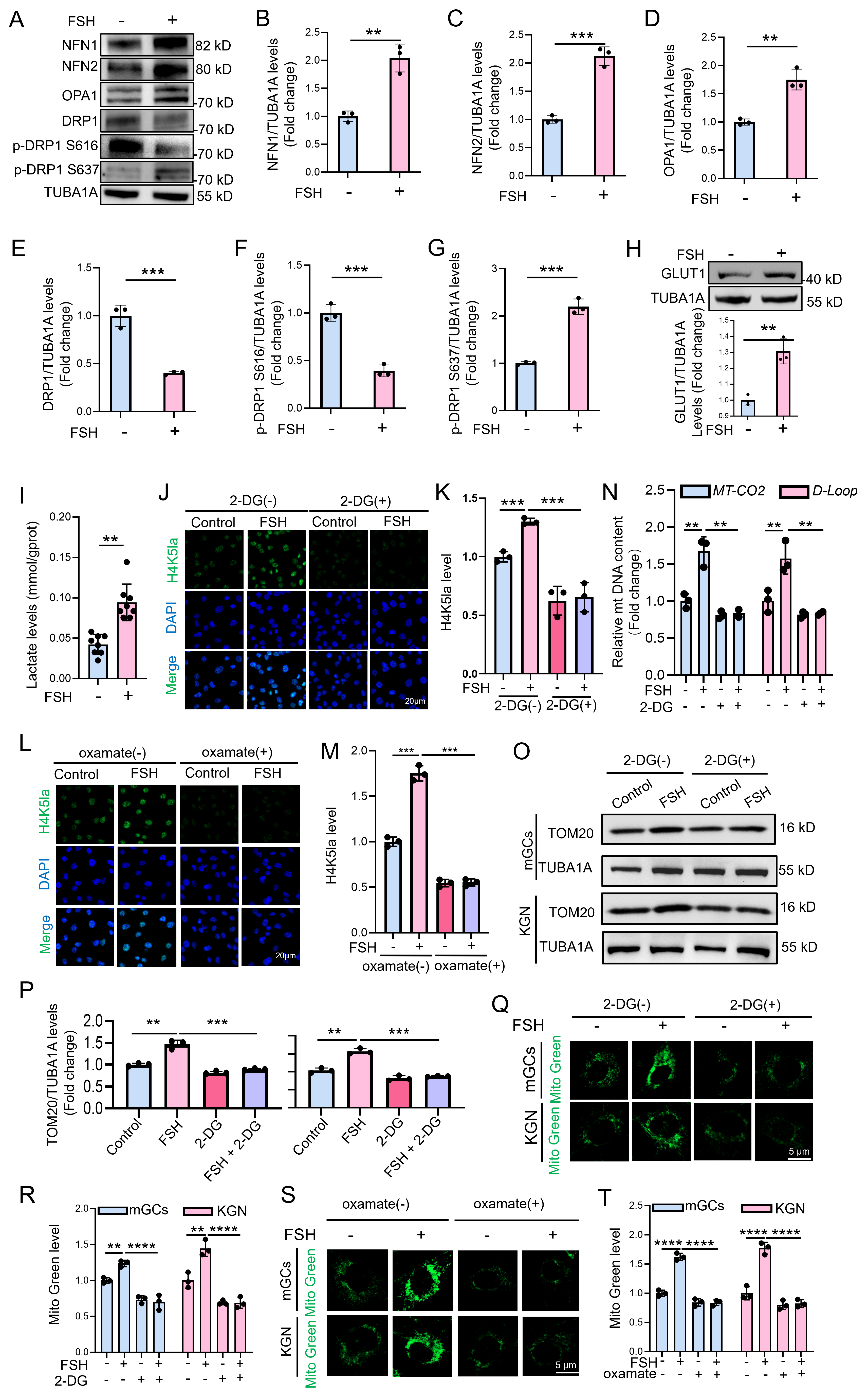
**

**Fig. S1. Inhibition of lactate production suppresses FSH-induced H4K5 lactylation and mitochondrial biogenesis.**

1. Analysis of mitochondrial fusion/fission protein expression by Western blot. (B - G) Quantitative analysis of mitochondrial fusion/fission related protein expression levels in (A), normalized to TUBA1A. (H) GLUT1 expression in mGCs in response to intraperitoneal FSH administration was quantified by western blot and normalized to TUBA1A. (I) Measurement of lactate levels in mGCs according to the FSH administration. Protein concentration served as the normalization control. (J) KGN cells were treated with 10 mM 2-DG for 2 h, followed by 5 IU FSH for 12 h. Immunofluorescence detection of H4K5la expression. (K) Quantitative analysis of H4K5la fluorescence intensity in (J). (L) KGN cells were treated with 10 mM oxamate for 2 h, followed by 5 IU FSH for 12 h. Immunofluorescence detection of H4K5la expression. (M) Quantitative analysis of H4K5la fluorescence intensity in (L). (N) RT-qPCR analysis of mitochondrial DNA copy number (*MT-CO2* and *D-Loop*) in KGN cells treated with 10 mM 2-DG for 2 h, followed by 5 IU FSH treatment for 12 h. *β-Actin* served as the loading control for data normalization. (O) mGCs and KGN cells were treated with 10 mM 2-DG for 2 h, followed by 5 IU FSH for 12 h. Western blot analysis of TOM20 protein levels. (P) Quantitative analysis of TOM20 protein expression levels in ( O), normalized to TUBA1A. (Q) mGCs and KGN cells were treated with 10 mM 2-DG for 2 h, followed by 5 IU FSH for 12 h. Mitochondria were stained with Mito Green (green) and observed using laser confocal scanning microscopy. Scale bar: 5 μm. (R) Quantitative analysis of Mito Green (green) fluorescence intensity in (Q). (S) mGCs and KGN cells were treated with 10 mM oxamate for 2 h, followed by 5 IU FSH for 12 h. Mitochondria were stained with Mito Green (green) and observed using laser confocal scanning microscopy. Scale bar: 5 μm. (T) Quantitative analysis of Mito Green fluorescence intensity in (S). Data are presented as the mean ± SD from at least three independent experiments (n ≥ 3). Statistical differences between groups were compared by one-way ANOVA followed by LSD post-hoc test.

**
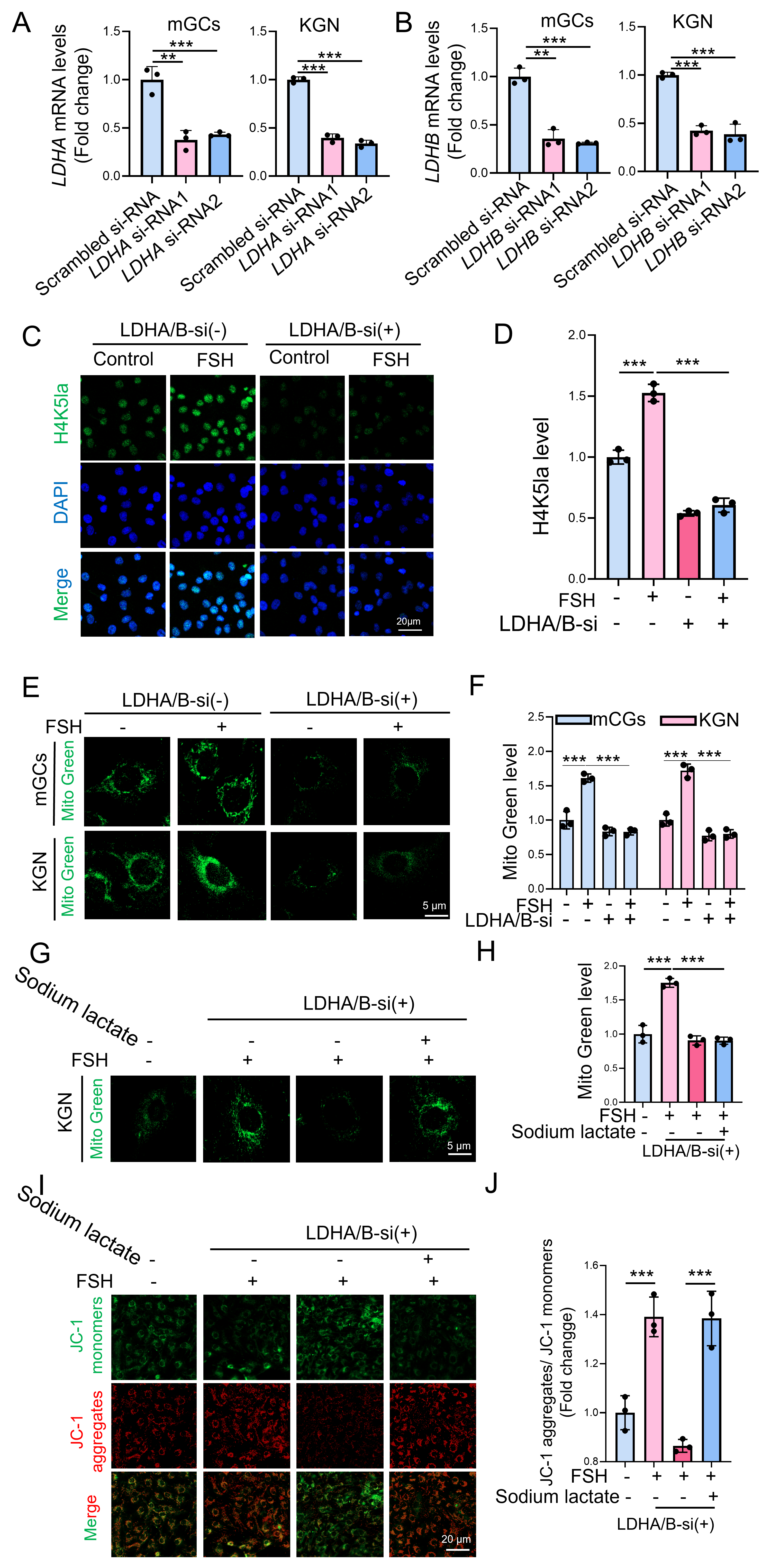
**

**Fig. S2. Knockdown of LDHA/B suppresses H4K5 lactylation and mitochondrial biogenesis.**

(A) Analysis of *LDHA* knockdown efficiency by RT-qPCR, normalized to *Tuba1a*. (B) Analysis of *LDHB* knockdown efficiency by RT-qPCR, normalized to *Tuba1a*. (C) Immunofluorescence analysis of H4K5la protein levels in KGN cells transfected with siRNAs targeting LDHA and LDHB for 12 h, followed by 5 IU FSH treatment for 12 h. Scale bar: 20 μm. (D) Quantitative analysis of H4K5la fluorescence intensity in (C). (E) KGN cells transfected with siRNAs targeting LDHA and LDHB for 12 h, followed by 5 IU FSH treatment for 12 h. Mitochondria were stained with Mito Green (green) and observed using laser confocal scanning microscopy. Scale bar: 5 μm. (F) Quantitative analysis of Mito Green fluorescence intensity in (E). (G) After transfection with LDHA/LDHB siRNAs for 12 h, KGN cells were exposed to 5 IU FSH for 12 h with or without 15 mM sodium lactate supplementation. Mitochondria were stained with Mito Green (green) and observed using laser confocal scanning microscopy. Scale bar: 5 μm. (H) Quantitative analysis of Mito Green fluorescence intensity in (G). (I) KGN cells underwent LDHA/LDHB siRNA transfection for 12 h followed by 12 h culture with 5 IU FSH, either supplemented with or without 15 mM sodium lactate. The mitochondrial membrane potential was assessed by JC-1 staining. (J) The membrane potential levels in (I) were analyzed. Data are presented as the mean ± SD from at least three independent experiments (n ≥ 3). Statistical differences between groups were compared by one-way ANOVA followed by LSD post-hoc test.


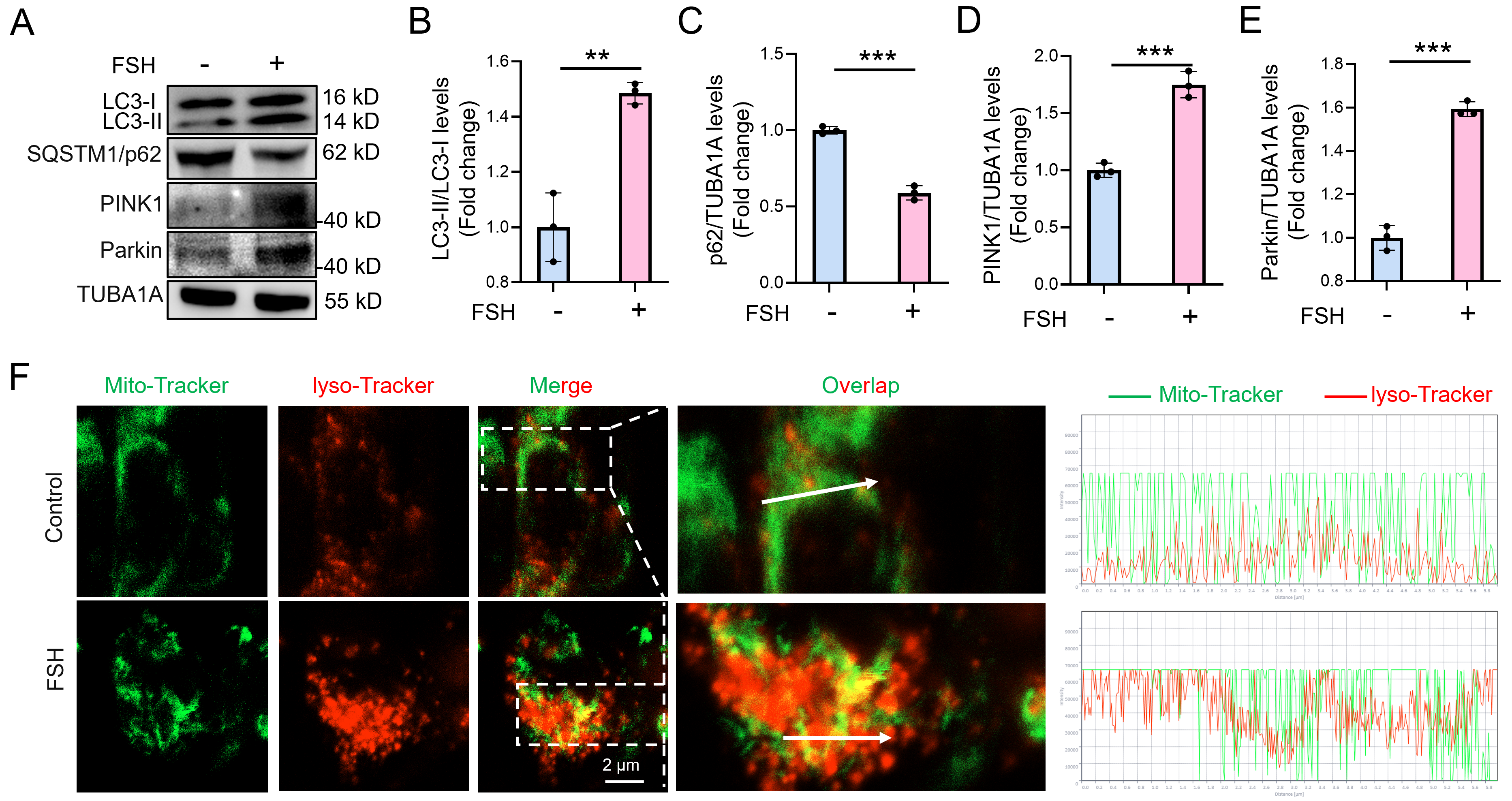


**Fig. S3. FSH promotes mitophagic activity.**

(A) Assessment of mitophagy-related protein expression levels by western blot after FSH treated for 12 h in KGN cells. (B - E) Quantitative analysis of mitophagy related protein expression levels in (A), normalized to TUBA1A. (F) After 12 h of FSH treatment, KGN cells were stained with MitoTracker and LysoTracker for 30 min, and colocalization of mitochondria and lysosomes was assessed by fluorescence microscopy. Scale bar: 2 μm. Data are presented as the mean ± SD from at least three independent experiments (n ≥ 3). Statistical differences between groups were compared by one-way ANOVA followed by LSD post-hoc test.


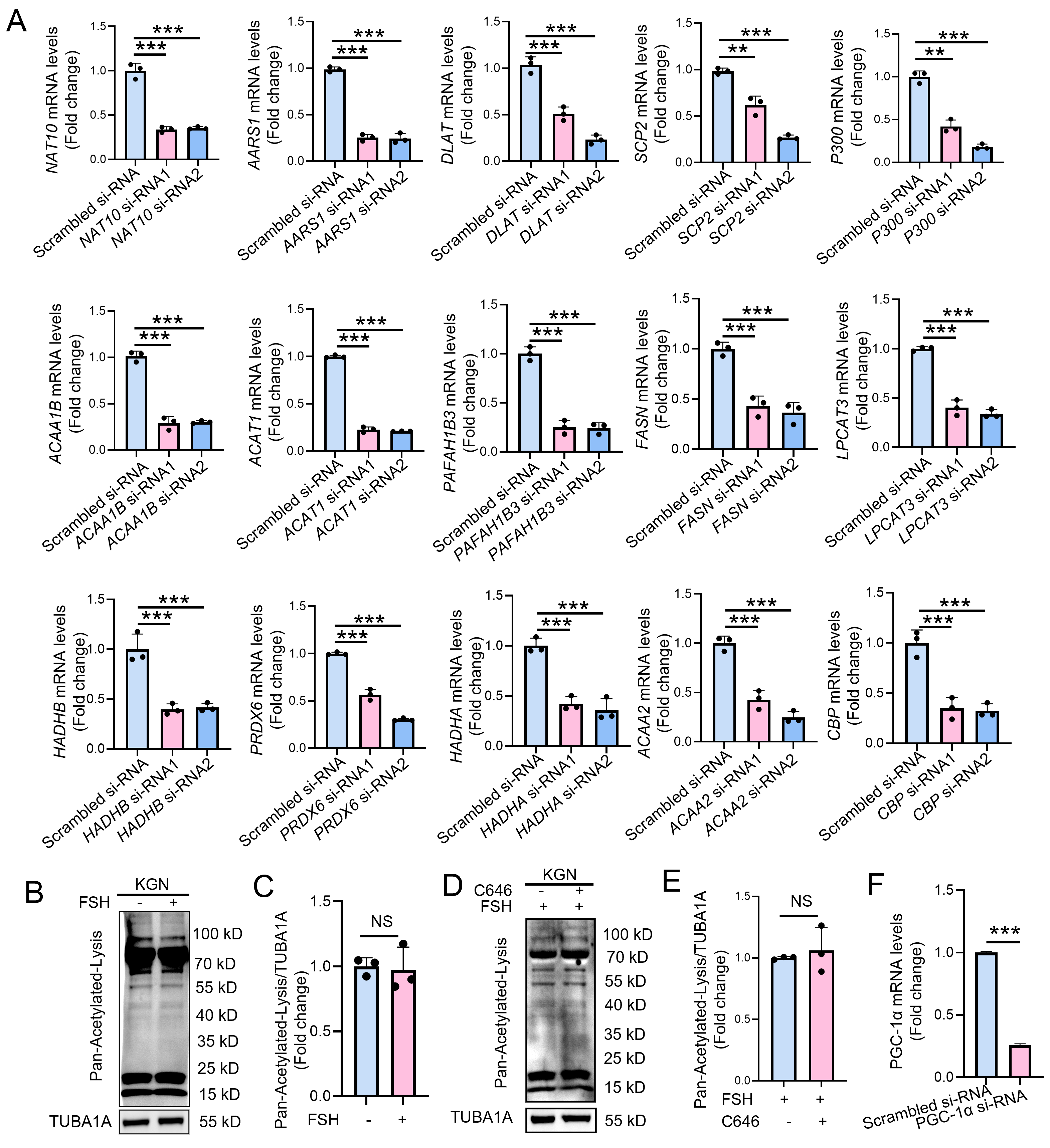


**Fig. S4. Assessment of acyltransferase knockdown efficiency.**

1. mRNA levels of various acyltransferases were assessed by RT–qPCR and normalized to *Tuba1a*. (B) Pan-Acetylated-Lysine levels after 12 h FSH treatment in KGN cells were analyzed by Western blot. (C) Quantitative analysis of Pan-Acetylated-Lysine levels in (B), normalized to TUBA1A. (D) Western blot of Pan-Acetylated-Lysine in KGN cells treated with 10 μM C646 for 2 h, followed by 5 IU FSH for 12 h. (E) Quantification of Pan-Acetylated-Lysine levels in (D), normalized to TUBA1A. (F) mRNA levels of PGC-1α were assessed by RT–qPCR and normalized to *Tuba1a*. Data are presented as the mean ± SD from at least three independent experiments (n ≥ 3). Statistical differences between groups were compared by one-way ANOVA followed by LSD post-hoc test.


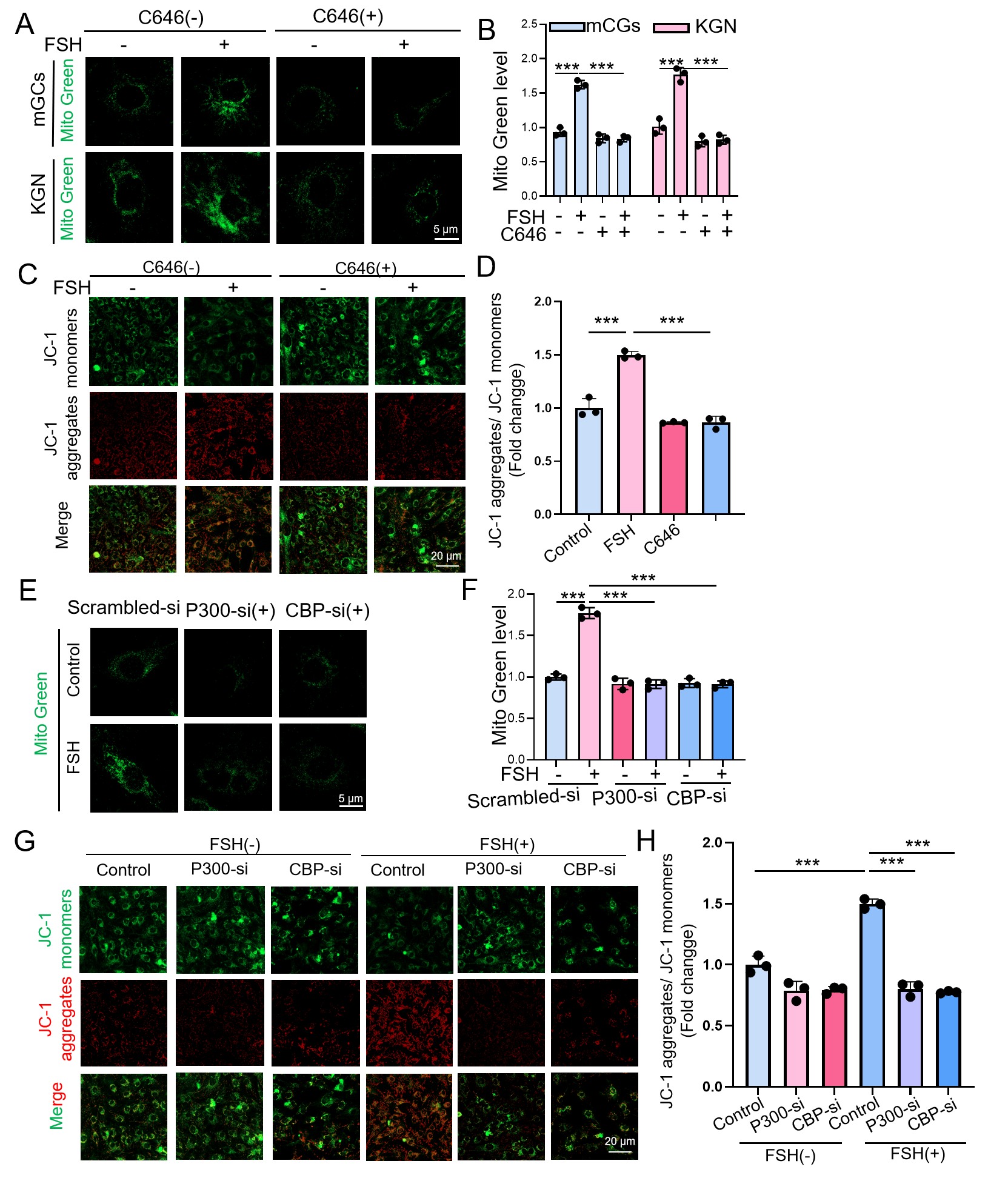


**Fig. S5.** Inhibition of P300/CBP blocks the FSH-induced increase in mitochondrial membrane potential.

(A) mGCs and KGN cells were treated with 10 μM C646 for 2 h, followed by 5 IU FSH for 12 h. Mitochondria were stained with Mito Green (green) and observed using laser confocal scanning microscopy. Scale bar: 5 μm. (B) Quantitative analysis of Mito Green (green) fluorescence intensity in (A). (C) KGN cells were treated with 10 μM C646 for 2 h, followed by 5 IU FSH for 12 h. JC-1 staining measured mitochondrial membrane potential. (D) The membrane potential levels in (C) were analyzed. (E) KGN cells received 12 h of P300 or CBP specific siRNAs transfection, then underwent 12 h of 5 IU FSH treatment. Mitochondria were visualized using MitoTracker Green and imaged by laser confocal scanning microscopy. Scale bar: 5 μm. (F) Quantitative analysis of Mito Green fluorescence intensity in (E). (G) KGN cells underwent P300 or CBP siRNA transfection for 12 h, then received 5 IU FSH for another 12 h. JC-1 staining measured mitochondrial membrane potential. (H) The membrane potential levels in (G) were analyzed. Data are presented as the mean ± SD from at least three independent experiments (n ≥ 3). Statistical differences between groups were compared by one-way ANOVA followed by LSD post-hoc test.


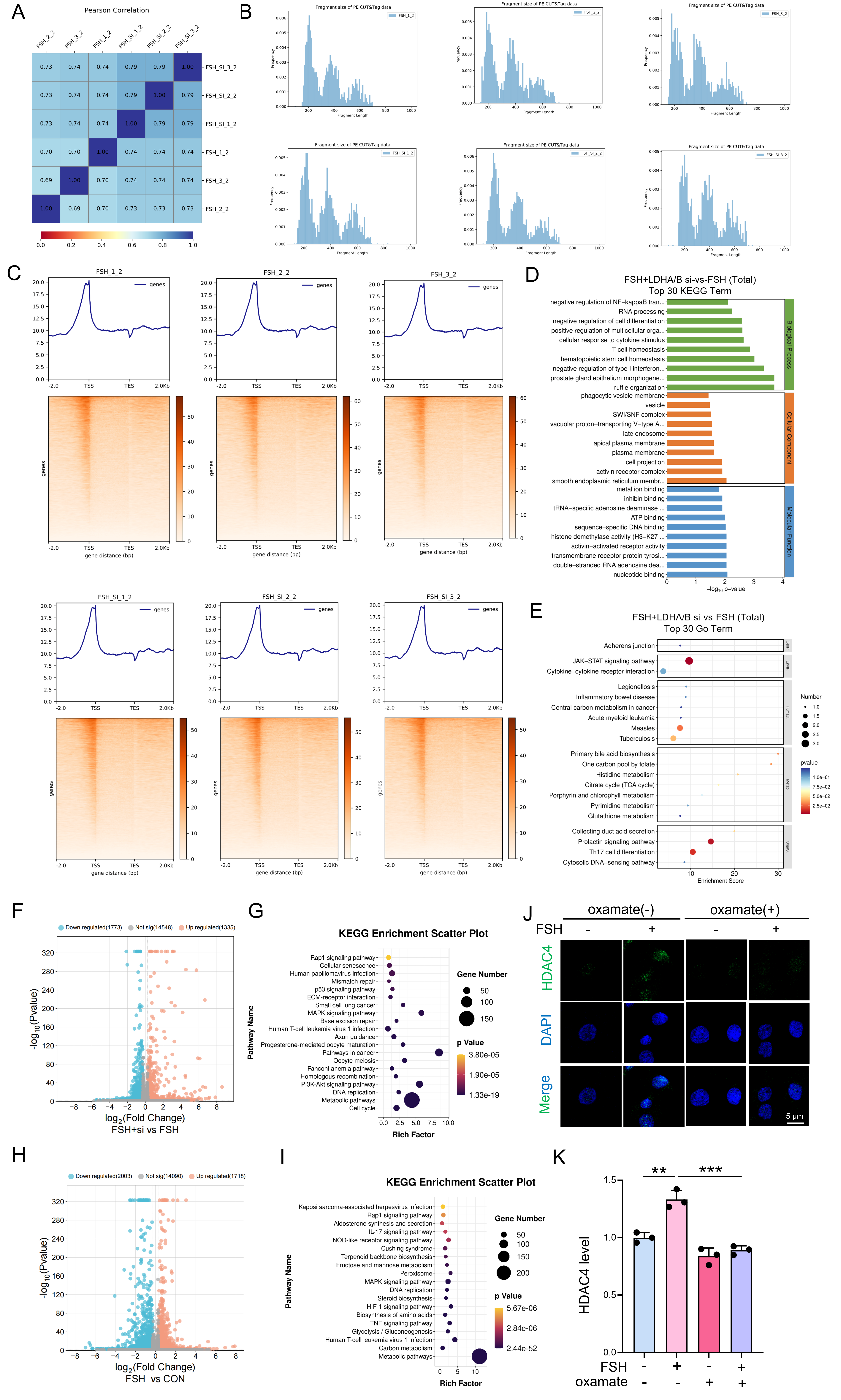


**Fig. S6. Diagram of differentially expressed genes.**

(A) Correlation analysis between individual samples in CUT&Tag analysis. (B) Assessment of CUT&Tag library quality by profiling insert size distributions. (C) CUT&Tag analysis was performed using H4K5la antibodies in mGCs following the indicated treatments, showing the distribution of H4K5la sites relative to the transcription start site (TSS). (D) Kyoto Encyclopedia of Genes and Genomes (KEGG) pathway analysis of H4K5la-targeted genes. (E) Gene Ontology (GO) analysis of H4K5la-targeted genes. (F) Analysis of the number of upregulated and downregulated genes following knockdown of LDHA and LDHB in the presence of FSH in mGCs. (G) KEGG pathway analysis of downregulated genes following knockdown of LDHA and LDHB in the presence of FSH in mGCs. (H) Analysis of the number of upregulated and downregulated genes in mGCs treated with FSH. (I) KEGG pathway analysis of upregulated genes in mGCs treated with FSH. (J) KGN cells were treated with 10 mM oxamate for 2 h, followed by 5 IU FSH for 12 h. Immunofluorescence detection of HDAC4 expression. (K) Quantitative analysis of HDAC4 fluorescence intensity in (J). Data are presented as the mean ± SD from at least three independent experiments (n ≥ 3). Statistical differences between groups were compared by one-way ANOVA followed by LSD post-hoc test.


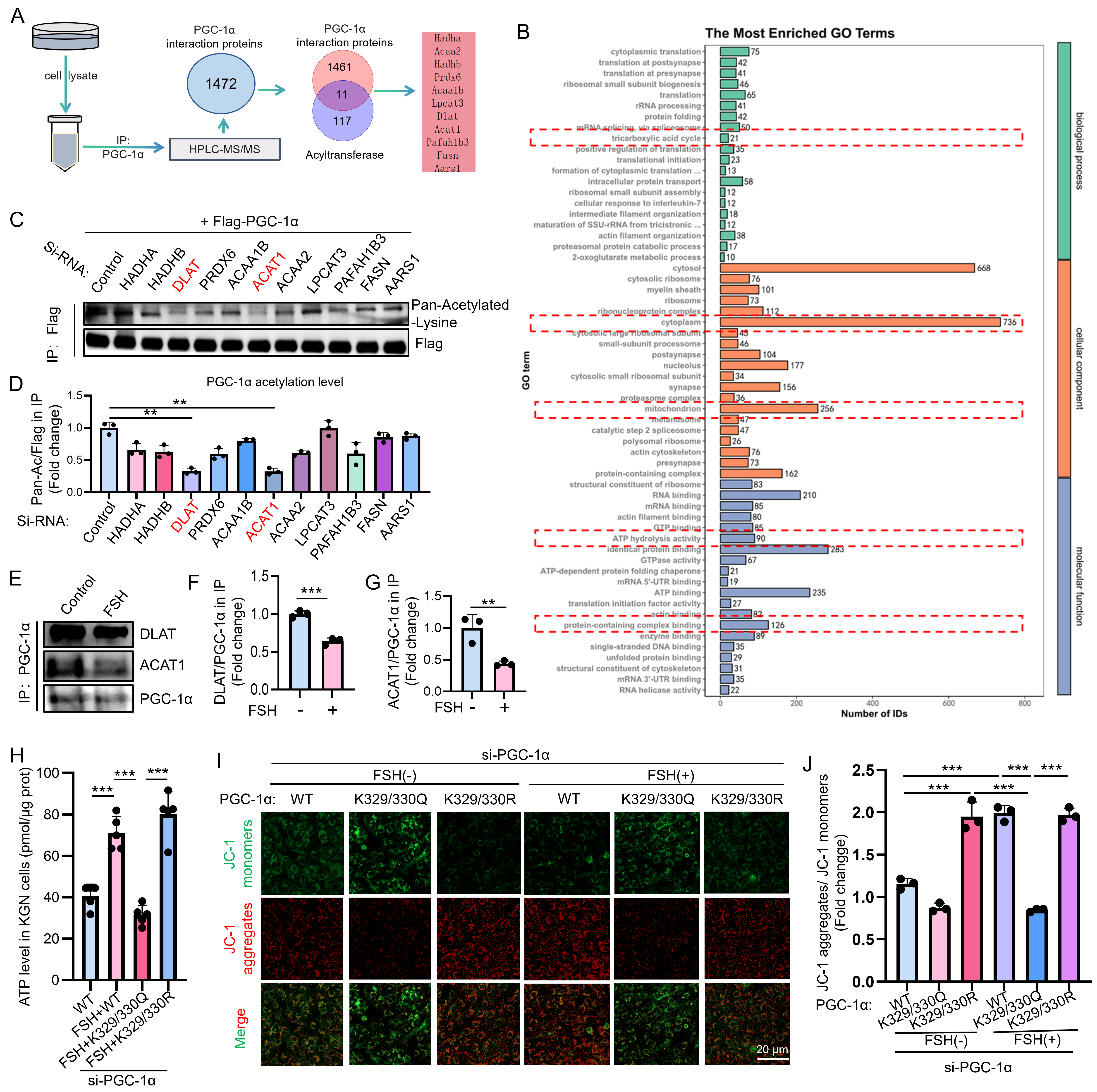


**Fig. S7. DLAT and ACAT1 are potential acetyltransferases for PGC-1α K329/K330 acetylation.**

(A) Illustration of PGC-1α-associated acetyltransferase detection via Immunoprecipitation followed by Mass Spectrometry. (B) GO analysis of the proteins showing interacted with PGC-1α. (C) Co-IP analysis of PGC-1α acetylation levels following acetyltransferase knockdown for 24 h. (D) Quantitative analysis of the acetylation modification level of PGC-1α in (C). (E) Assessment of PGC-1α interaction with DLAT and ACAT1 by Co-IP after FSH treated for 12 h. (F and G) Quantitative analysis of the binding levels of DLAT and ACAT1 to PGC-1α in (E). The interactions were quantified as the ratio of DLAT/PGC-1α and ACAT1/PGC-1α. (H) PGC-1α knockdown KGN cells overexpressing Flag-tagged wild-type (WT) PGC-1α, K329/330R PGC-1α (acetylation-resistant), or K329/330Q PGC-1α (acetylation-mimic) for 12 h, then 5 IU FSH for 12 h. The ATP level was measured. (I) PGC-1α knockdown KGN cells overexpressing Flag-tagged wild-type (WT) PGC-1α, K329/330R PGC-1α (acetylation-resistant), or K329/330Q PGC-1α (acetylation-mimic) exposed for 12 h, then treated with 5 IU FSH over 12 h. The mitochondrial membrane potential was assessed by JC-1 staining. (J) The membrane potential levels in (I) were analyzed. Data are presented as the mean ± SD from at least three independent experiments (n ≥ 3). Statistical differences between groups were compared by one-way ANOVA followed by LSD post-hoc test.

**
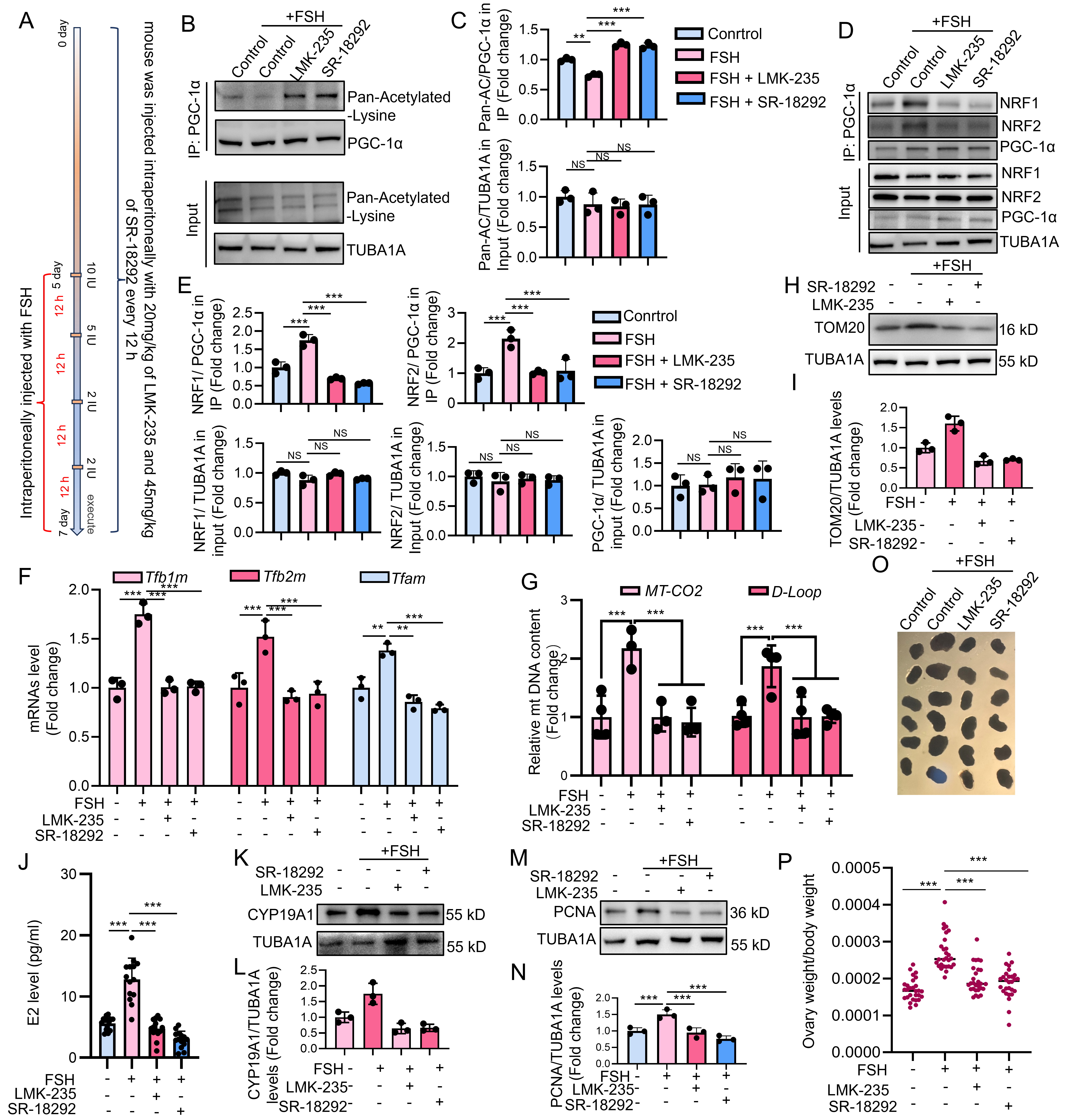
**

**Fig S8. In vivo validation of the mechanistic model through intraperitoneal injection of LMK-235 or SR-18292.**

(A) Schematic representation of the in vivo lactylation inhibition experimental workflow. (B) Co-IP analysis detecting the interaction between PGC-1α and pan-acetylated lysine following the indicated treatments in (A). (C) Quantitative analysis of the acetylation modification level of PGC-1α in (B). For the IP, PGC-1α acetylation was quantified as the ratio of Pan-Kla signal to PGC-1α signal. For the input, the acetylation level was normalized to TUBA1A. (D) Co-IP analysis detecting the interaction between PGC-1α and NRF1/2 following the indicated treatments in (A). (E) Quantitatively measure the binding affinity of PGC-1α to proteins NRF1 and NRF2 in (D). For the IP, the binding of PGC-1α to NRF1 and NRF2 was quantified as the ratio of NRF1/PGC-1α and NRF2/PGC-1α. For the input, the protein levels of PGC-1α, NRF1, and NRF2 were normalized to TUBA1A. (F) RT-qPCR analysis of *Tfb1m*, *Tfb2m*, and *Tfam* mRNA levels following the treatments indicated in panel (A), normalized to *Tuba1a*. (G) RT-qPCR analysis of mitochondrial DNA copy number (MT-CO2 and D-Loop) following the indicated treatments in (A). β-Actin served as the loading control for data normalization. (H) Western blot analysis of TOM20 protein levels following the indicated treatments in (A). (I) The protein levels of TOM20 in (H) was quantitatively analyzed with normalization to TUBA1A. (J) The levels of E2 in serum were measured by radioimmunoassay (RIA) following the indicated treatments in (A). (K) Western blot analysis of CYP19A1 protein levels following the indicated treatments in (A). (K) The protein levels of CYP19A1 in (K) was quantitatively analyzed with normalization to TUBA1A. (M) Western blot analysis of PCNA protein levels following the indicated treatments in (A). (N) The protein levels of PCNA in (M) was quantitatively analyzed with normalization to TUBA1A. (O) Ovarian size quantification under experimental conditions. (P) Ovarian weight measurement following indicated treatments. The ovary weight was expressed relative to the body weight of the corresponding mouse. Data are presented as the mean ± SD from at least three independent experiments (n ≥ 3). Statistical differences between groups were compared by one-way ANOVA followed by LSD post-hoc test.

**Supplementary Table S1.** Primer sequences for RT-qPCR.

| *HDAC4-mus* | forward primer | GCCCTGCCGCTGGTG |
| --- | --- | --- |
|  | reverse primer | AAGGGGTGTCTGGGTGGG |
| *TUBA1A-mus* | forward primer | AAGCAGCAACCATGCGTGA |
|  | reverse primer | CCTCCCCCAATGGTCTTGTC |
| *HDAC4-HOMO* | forward primer | TGGAGCTATCGTTTCCGTGG |
|  | reverse primer | GCCACATCCACCGTGCT |
| *TUBA1A-HOMO* | forward primer | TTCTGTCTCGCGTTGTTCTCT |
|  | reverse primer | GATGCACTCACGCATGTTTT |
| *LDHA-mus* | forward primer | TGGGCTCCCACTCTGACG |
|  | reverse primer | GACTTTGAATCTTTTGAGACCGCT |
| *LDHB-mus* | forward primer | GGTGGTGGACAGGGAATGTA |
|  | reverse primer | TTTCTGGATGTCCCACAGGG |
| *LDHA-HOMO* | forward primer | TTGTCTCTGGCAAAGTGGAT |
|  | reverse primer | CTCCATGTTCCCCAAGGACC |
| *LDHB-HOMO* | forward primer | TTAGCTCTGAGCATCCGGC |
|  | reverse primer | GCCATTTTGCACAAGGACA |
| *NAT10* | forward primer | CAGTCGGCTCTTCTCTTG |
|  | reverse primer | CATCCAGGTCATCACTCAG |
| *AARS1* | forward primer | GCTGTTTATTGATGAGCCCCG |
|  | reverse primer | TCAGGAGCAACCAGTGAACC |
| *DLAT* | forward primer | CAGAGATAGAGACCGACAAG |
|  | reverse primer | GCAAGAGGACTAACGAACA |
| *SCP2* | forward primer | ACAAGAATTATGGTCAGAGC |
|  | reverse primer | GCAGACAATCAAGTGTATGA |
| *P300* | forward primer | CGCAGCTTCTAGGAATCCCTGATTA |
|  | reverse primer | CAGGGCTCTTGGTATTGTCCA |
| *ACAA1B* | forward primer | GCATCCAATCCGGTTCTC |
|  | reverse primer | CCATCACATCCACCATTCA |
| *ACAT1* | forward primer | AAGACAGCCAGGACTACA |
|  | reverse primer | CCAACACTACCGACTGAG |
| *PAFAH1B3* | forward primer | ACAGCAGAACAAGTGACAG |
|  | reverse primer | GAGACGAAGAAGCAAGGAG |
| *FASN* | forward primer | CTGCCTTCGGTTCAGTCTCTT |
|  | reverse primer | CACCCTCCAAGGAGTCTCAC |
| *LPCAT3* | forward primer | TCAACTTTGGCCTACCTTCTTG |
|  | reverse primer | CAGGTATGGTGCCCGTCTT |
| *HADHB* | forward primer | CAGCTGGAGGACAGGGTCAT |
|  | reverse primer | AGAGATCAGTCGGTCGCCTC |
| *PRDX6* | forward primer | CTTGTTCTCAGCGTCACCAC |
|  | reverse primer | CTGTCTCCTCAGCAGGAATCT |
| *HADHA* | forward primer | CAAGGGCTTCTTAGGTCGCA |
|  | reverse primer | GCAGCCTCAGATTTGCCAAG |
| *ACAA2* | forward primer | GGTTCATGAGTTAAGGCGTCG |
|  | reverse primer | AAGGTCTCCTGTGTTCCGTG |
| *CREBBP* | forward primer | CAGCAGCAGCACCTATAC |
|  | reverse primer | TGGCATACTAGACATGACAG |

**Supplementary Table S2.** Primer sequences for ChIP-PCR.

| *TFAM* | forward primer | TGCGGTTTCCCTTCATCTCC |
| --- | --- | --- |
|  | reverse primer | CACTAGCGAGGCACTATGGG |
| *TFB1M* | forward primer | CTCTCACAGGCGGAAGCC |
|  | reverse primer | CTCGGAGTCAGCCCCATTG |
| *TFB2M* | forward primer | CCTCAACGGTCCACTCACAA |
|  | reverse primer | CGTGGAACATTTTCTGGCGT |
| *HDAC4* | forward primer | ACAGGGGAGCTGAAGAATGG |
|  | reverse primer | GAGGATCTTGCTCACGCTCA |

**Supplementary Table S3.** Primer sequences of Mitochondrial DNA copy number for qRT-PCR.

| *D-Loop* | forward primer | GATTTGGGTACCACCCAAGTATTG |
| --- | --- | --- |
|  | reverse primer | GTACAATATTCATGGTGGCTGGCA |
| *MT-CO2* | forward primer | CCTGCGACTCCTTGACGTTG |
|  | reverse primer | AGCGGTGAAAGTGGTTTGGTT |
| *β-actin* | forward primer | TCACCCACACTGTGCCCATCTACGA |
|  | reverse primer | CAGCGGAACCGCTCATTGCCAATGG |

**Supplementary Table S4.** siRNA sequences

| *Scrambled siRNA* | Sense (5'-3') | UUCUCCGAACGUGUCACGUTT |
| --- | --- | --- |
|  | Antisense (5'-3') | ACGUGACACGUUCGGAGAATT |
| *NAT10-siRNA1* | Sense (5'-3') | UUGCUGUUCACCCAGAUUAUCTT |
|  | Antisense (5'-3') | GAUAAUCUGGGUGAACAGCAATT |
| *NAT10-siRNA2* | Sense (5'-3') | GCAGUGGAGAAGUGGCUUAAUTT |
|  | Antisense (5'-3') | AUUAAGCCACUUCUCCACUGCTT |
| *AARS1-siRNA1* | Sense (5'-3') | CCUCGUGUUCAUCCAGUAUAATT |
|  | Antisense (5'-3') | UUAUACUGGAUGAACACGAGGTT |
| *AARS1-siRNA2* | Sense (5'-3') | GCUGCACAUAGGAACGAUAUATT |
|  | Antisense (5'-3') | UAUAUCGUUCCUAUGUGCAGCTT |
| *DLAT-siRNA1* | Sense (5'-3') | GCAGAGGUUGAAACUGAUAAATT |
|  | Antisense (5'-3') | UUUAUCAGUUUCAACCUCUGCTT |
| *DLAT-siRNA2* | Sense (5'-3') | CCGCAUCAGAAGGUUCCAUUATT |
|  | Antisense (5'-3') | UAAUGGAACCUUCUGAUGCGGTT |
| *SCP2-siRNA1* | Sense (5'-3') | GUUGGCUAUGAUAUGAGUAAATT |
|  | Antisense (5'-3') | UUUACUCAUAUCAUAGCCAACTT |
| *SCP2-siRNA2* | Sense (5'-3') | UAGUGCCUCACACUCAAUUACTT |
|  | Antisense (5'-3') | GUAAUUGAGUGUGAGGCACUATT |
| *P300-siRNA1* | Sense (5'-3') | CCGGUGAACUCUCCUAUAATT |
|  | Antisense (5'-3') | UUAUAGGAGAGUUCACCGGTT |
| *P300-siRNA2* | Sense (5'-3') | GCCUCAAACUACAAUAAAUTT |
|  | Antisense (5'-3') | AUUUAUUGUAGUUUGAGGCTT |
| *ACAA1B-siRNA1* | Sense (5'-3') | GAGGGAACCAUGGGAAUAUUUTT |
|  | Antisense (5'-3') | AAAUAUUCCCAUGGUUCCCUCTT |
| *ACAA1B-siRNA2* | Sense (5'-3') | GUGUCACAGCACUUUAAUUUATT |
|  | Antisense (5'-3') | UAAAUUAAAGUGCUGUGACACTT |
| *ACAT1-siRNA1* | Sense (5'-3') | GUUCGGUCUGGCUAGUAUUUGTT |
|  | Antisense (5'-3') | CAAAUACUAGCCAGACCGAACTT |
| *ACAT1-siRNA2* | Sense (5'-3') | GGGCGCAGGUUUACCUAUUUCTT |
|  | Antisense (5'-3') | GAAAUAGGUAAACCUGCGCCCTT |
| *PAFAH1B3-siRNA1* | Sense (5'-3') | GUGCAUUCUGAUGGCACCAUATT |
|  | Antisense (5'-3') | UAUGGUGCCAUCAGAAUGCACTT |
| *PAFAH1B3-siRNA2* | Sense (5'-3') | CGGUUUGUAGCGGACAGCAAATT |
|  | Antisense (5'-3') | UUUGCUGUCCGCUACAAACCGTT |
| *FASN-siRNA1* | Sense (5'-3') | GCUGGUCGUUUCUCCAUUAAATT |
|  | Antisense (5'-3') | UUUAAUGGAGAAACGACCAGCTT |
| *FASN-siRNA2* | Sense (5'-3') | GCUGCGGAAACUUCAGGAAAUTT |
|  | Antisense (5'-3') | AUUUCCUGAAGUUUCCGCAGCTT |
| *LPCAT3-siRNA1* | Sense (5'-3') | CGAGGAUCUGAGCCUUAACAATT |
|  | Antisense (5'-3') | UUGUUAAGGCUCAGAUCCUCGTT |
| *LPCAT3-siRNA2* | Sense (5'-3') | CCUACUAUUCAUAUUGCCUUATT |
|  | Antisense (5'-3') | UAAGGCAAUAUGAAUAGUAGGTT |
| *HADHB-siRNA1* | Sense (5'-3') | CCUAUUCGUCAUUCAAGAAAUTT |
|  | Antisense (5'-3') | AUUUCUUGAAUGACGAAUAGGTT |
| *HADHB-siRNA2* | Sense (5'-3') | CAUGGCUUGUAUCUCUUCAAATT |
|  | Antisense (5'-3') | UUUGAAGAGAUACAAGCCAUGTT |
| *PRDX6-siRNA1* | Sense (5'-3') | GGACGCUAACAACAUGCCUGU |
|  | Antisense (5'-3') | AGGCAUGUUGUUAGCGUCCUU |
| *PRDX6-siRNA2* | Sense (5'-3') | GAUCGUGGCAUGAUCACAGCC |
|  | Antisense (5'-3') | CUGUGAUCAUGCCACGAUCUU |
| *HADHA-siRNA1* | Sense (5'-3') | GCUGACCAGAACCCAUAUUAATT |
|  | Antisense (5'-3') | UUAAUAUGGGUUCUGGUCAGCTT |
| *HADHA-siRNA2* | Sense (5'-3') | UGCUGACCAGAACCCAUAUUATT |
|  | Antisense (5'-3') | UAAUAUGGGUUCUGGUCAGCATT |
| *ACAA2-siRNA1* | Sense (5'-3') | CACACCUGGUUCAUGAGUUAATT |
|  | Antisense (5'-3') | UUAACUCAUGAACCAGGUGUGTT |
| *ACAA2-siRNA2* | Sense (5'-3') | UACUUUGUGGGCAGGAUUAACTT |
|  | Antisense (5'-3') | GUUAAUCCUGCCCACAAAGUATT |
| *CBP-siRNA1* | Sense (5'-3') | CUGGCCAUGCUGGACUAAAUATT |
|  | Antisense (5'-3') | UAUUUAGUCCAGCAUGGCCAGTT |
| *CNP-siRNA2* | Sense (5'-3') | UCCUAGGAAUCCCAGAUUAUUTT |
|  | Antisense (5'-3') | AAUAAUCUGGGAUUCCUAGGATT |
| *LDHA-siRNA1*  (*HOMO*) | Sense (5'-3') | CUGGCAAAGACUAUAAUGUTT |
|  | Antisense (5'-3') | ACAUUAUAGUCUUUGCCAGTT |
| *LDHA-siRNA2*  (*HOMO*) | Sense (5'-3') | CGGUUGCAAUCUGGAUUCAT |
|  | Antisense (5'-3') | CGGUUGCAAUCUGGAUUCAT |
| *LDHA-siRNA1*  (*Mus*) | Sense (5'-3') | CCAGUUUCCACCAUGAUUATT |
|  | Antisense (5'-3') | UAAUCAUGGUGGAAACUGGTT |
| *LDHA-siRNA2*  (*Mus*) | Sense (5'-3') | GGGUCUCUAUGGAAUCAAUTT |
|  | Antisense (5'-3') | AUUGAUUCCAUAGAGACCCTT |
| *LDHB-siRNA1*  (*HOMO*) | Sense (5'-3') | CGUGUGCUAUCAGCAUUCUTT |
|  | Antisense (5'-3') | AGAAUGCUGAUAGCACACGTT |
| *LDHB-siRNA2*  (*HOMO*) | Sense (5'-3') | CUGGUGCAGAGAAAUGUUATT |
|  | Antisense (5'-3') | UAACAUUUCUCUGCACCAGTT |
| *LDHB-siRNA1*  (*Mus*) | Sense (5'-3') | CUGGGCUAUUGGAUUAAGUTT |
|  | Antisense (5'-3') | ACUUAAUCCAAUAGCCCAGTT |
| *LDHB-siRNA2*  (*Mus*) | Sense (5'-3') | GACAAGCUCAAAGGAGAGATT |
|  | Antisense (5'-3') | UCUCUCCUUUGAGCUUGUCTT |
| *HDAC4-siRNA1* | Sense (5'-3') | CACAGUUGCAUGAACAUAUTT |
|  | Antisense (5'-3') | AUAUGUUCAUGCAACUGUGTT |
| *HDAC4-siRNA2* | Sense (5'-3') | CACCAUCCUUACCCAACAUTT |
|  | Antisense (5'-3') | AUGUUGGGUAAGGAUGGUGTT |
| *HDAC4-siRNA3* | Sense (5'-3') | GCGACACCAUAUGGAAUGATT |
|  | Antisense (5'-3') | GCGACACCAUAUGGAAUGATT |
| *HDAC4-siRNA4* | Sense (5'-3') | GGCUGAAUGUGAGCAAGAUTT |
|  | Antisense (5'-3') | AUCUUGCUCACAUUCAGCCTT |
| *PGC-1α-siRNA* | Sense (5'-3') | GACGACGAAGCAGACAAGATT |
|  | Antisense (5'-3') | UCUUGUCUGCUUCGUCGUCTT |
